# Superoxide dismutase (SOD) as a selection criterion for triticale grain yield under drought stress: a comprehensive study on genomics and expression profiling, bioinformatics, heritability, and phenotypic variability

**DOI:** 10.1186/s12870-021-02919-5

**Published:** 2021-03-22

**Authors:** Armin Saed-Moucheshi, Fatemeh Sohrabi, Elham Fasihfar, Fatemeh Baniasadi, Mehrnaz Riasat, Ali Akbar Mozafari

**Affiliations:** 1 Department of Horticulture, Faculty of Agriculture, University of Kurdistan, Sanandaj, Iran; 2 Horticulture and Crop Research Department, Kermanshah Agricultural and Natural Resources Research and Education Center (AREEO), Kermanshah, Iran; 3 Department of Plant Biotechnology, School of Agriculture, Shiraz University, Shiraz, Iran; 4 Department of Plant Pathology, Faculty of Agriculture, Tarbiat Modares University, Tehran, Iran; 5 Department of Horticultural Science, Faculty of Agriculture, University of Zanjan, Zanjan, Iran; 6 Department of Natural Resources, Researches and Education Center of Agriculture and Natural Resources of Fars Province, Shiraz, Iran; 7 Agricultural Research, Education and Extension Organization (AREEO), Tehran, Iran

**Keywords:** Heatmap, Mn-SOD, Cu/Zn-SOD, Fe-SOD, Promotor analysis, Feature selection

## Abstract

**Background:**

The main objectives of this study were to find the possible structural association between the activity of enzymatic antioxidants and the grain yield of triticale plants as well as identifying the genotypic variability which might be effective on this association. Accordingly, expression levels of superoxide dismutase (SOD) isozymes (Mn-SOD, Cu/Zn-SOD, and Fe-SOD) were appraised to distinguish any possible relationship between SOD expression and drought resistance of triticale. A novel analytical method for distinguishing elite genotypes based on measured features was proposed. Additionally, a new programing based on SAS-language (IML) was introduced to estimate the genetic parameters rooted from combined ANOVA model (linear mixed model), which is capable of being used in any field study other than the current one.

**Methods:**

Thirty genotypes of triticale were studied under normal and drought stress conditions during 6 years (three different locations). Accordingly, based on the results of genetic variability, heatmap analysis, biplot graph, and clustering technique, two genotypes with the highest genetic distance were selected to appraise the differential expression profiling of three SOD isozyme in shoot and root organs.

**Results:**

Field experiments and bioinformatics results showed that superoxide dismutase (SOD) was the most influential antioxidant in resistance of triticale to drought stress; therefore, it could be used as an indirect selection index in early stages to distinguish resistant genotypes to drought stress. Additionally, Mn-SOD and Fe-SOD showed roughly similar expression levels for both genotypes under drought stress. However, Cu/Zn-SOD expression level was higher in root and shoot of the tolerant genotype than the susceptible genotype.

**Conclusion:**

Heatmap analysis that is applied for the first time to screen suitable genotypes, showed to be highly capable of distinguishing elite genotypes and pointing out the proper features for selection criteria. Bioinformatics results indicated that SOD is more important than other enzymatic antioxidant for being considered as selection criteria or candidate gene for transgenic purposes. Based on expressional results, Mn-SOD announced as a general isozyme that is probably highly expressed in most of the species, while, Cu/Zn-SOD was introduced as a genotype specific isozyme that is likely more expressed in tolerant genotypes

**Supplementary Information:**

The online version contains supplementary material available at 10.1186/s12870-021-02919-5.

## Background

High demands for agricultural products as a result of growing population has led researchers to apply and review altered procedures and approaches so as to increase the production and adoptability of the crops [56]. Many efforts have been done in order to combine varied capabilities of different plant species into one unique plant. Subsequently, over 100 years ago scientists had been able to made and release a new plant species by crossing between wheat (*Triticum sp.*) and rye (*Secale cereale*) plants [10]. The intention was to increase the capability of wheat, as one of the most significant sources of food amongst cereals in the world, to resist the harsh environmental conditions such as drought stress. Different crossing between rye and wheat with altered polyploidy levels resulted in different types of plants with different properties inherited from different genome sets [53]. Triticale (×*Triticosecale Wittmack*) is a hexaploidy species (AABBRR) resulted from crossing between tetraploid wheat (*T. durum* Desf; 2n = 28: AABB), known as durum wheat, and diploid rye (2n = 14: RR). Final release of triticale inherited favorable features and properties from both of its progenitors such as higher resistance to drought stress and higher plant production in comparison to wheat plants [37].

Resistance to drought stress is a complex process involving different types of physiological and molecular networks and responses which has not been properly unveiled and addressed yet [40, 57]. Drought stress, similar to the most of other environmental stresses, indirectly causes oxidative stress in plants as a result of disturbing the balance between production and detoxification of oxygen radicals [7, 11, 48]. In such condition, defense systems and stress resistance mechanisms in plants are activated to induce higher detoxification of oxygen radicals [27, 54]. Accordingly, the content of antioxidants in plant cells are increases as the results of activating and/or enhancing the expression profiling of related genes [26, 47, 50]. Superoxide dismutase (SOD) is family of genes that its enzymatic products are able to actively dismutase the superoxide radicals (O_2_^−^) in different plants’ organelles [17, 23]. According to numerus scientific reports, SOD family contains three main members i.e. magnesium-SOD (Mn-SOD), copper/zinc-SOD (Cu/Zn-SOD), and iron-SOD (Fe-SOD). Many studies have substantiated altered expression rates for each of the SOD isozymes under varies situation and stresses in different plants species [3, 20, 48] and even different genotypes within a species [12, 17, 54]. Therefore, delving over the SOD expressional mechanisms that are active in its responsive pathways along with identification of related regulatory elements may provide new insights toward understanding and recognizing the resistance mechanisms in plants. Additionally, finding the true association between SOD expression rate and plant productivity under drought stress condition would help breeders to identify resistant genotypes with more simplicity and increase the efficiency of breeding programs [34].

The main objectives of the current study were to find the structural association between the activity of enzymatic antioxidants and grain yield of triticale plants along with identify the genotypic variability of SOD activity among different triticale elite genotypes under normal irrigation and drought stress conditions by means of advanced and novel statistical methods. Also, the promotor analyzing of enzymatic antioxidants: SOD, glutathione reductase (GR), peroxidase (POD), catalase (CAT), and ascorbic peroxidase (APX) was carried out to evaluate the variation in regulatory network among these antioxidants in line with identify the key regulatory elements in these pathways. In addition, the difference between expression rate of SOD isozymes (Mn-SOD, Cu/Zn-SOD, and Fe-SOD) in selected triticale genotypes, with highest genetic distance and lowest similarity, were tested to find out about the relationship between their expression rate and the resistance of triticale plants to drought stress. The tolerant genotypes of triticale maintaining high production under drought stress condition, were then introduced as genotypes candidates for being used as new and favorable cultivars.

## Results

### ANOVA and genetic variability

The first three out of six years of the field study were performed in one location (Shiraz), the fourth year in another location (Zarghan), and the last two years in a separate location (Sanandaj); therefore, the effect of location, as a source of variance, in combined ANOVA is actually nested inside the effect of year that makes it impossible to separate these two effects from each other. Subsequently, the effect of year, as a representative of both year and location, was considered along with the effect of condition (stress), normal irrigation or drought stress condition within each year, and the effect of genotype in final ANOVA model as the main source of variation (Table 3). The effect of year was statistically significant (*p* < 0.05) for all measured features in triticale plants consisting of H_2_O_2_, MDH, chlorophyll, carotenoid, protein, proline, POD, CAT, APX, GR, SOD, and grain yield which indicates the overall difference between years and locations regarding these features. Similarly, there was significant differences between normal irrigation and drought stress conditions regarding all features in Table 3. However, no significant effect of stress × year interaction was found for chlorophyll content, protein content, POD and APX activities. Save for proline content, the main effect of genotype along with the interaction effects of year × genotype, stress × genotype, and stress × year × genotype were statistically significant for all other measured features (Supplementary Table 3).

Genetic parameters were estimated by taking year and genotype as random effects and calculating the expected values for each source of variation in ANOVA mixed model separately in each stress condition (Table 1). Accordingly, environmental, genotypic, and phenotypic variance were extracted from data and transformed into standard indices i.e. heritability, phenotypic coefficient of variation (PCV), and genotypic coefficient of variation (GCV). The highest heritability under normal irrigation and drought stress conditions were observed in carotenoid content (62%) and SOD activity (61%), respectively. The protein content showed the lowest percentage of heritability in both normal irrigation (6.81%) and drought stress conditions (4.15%). Under normal irrigation condition, proline content had the highest PCV (70%) and GR activity showed the highest GCV (26.6%). The lowest PCV (7.5%) and GCV (1.6%), on the other side, were obtained for H_2_O_2_ in normal irrigation condition. MDH and CAT activity were the feature with the same lowest percentage of PCV (33.6%) in drought stress condition. The highest PCV percentage under drought stress condition was observed in APX activity. SOD activity showed the highest GCV (46.4%) in drought stress condition, while the lowest in this condition was estimated for protein content (2.19%).

**Table 1:** Genetic parameters estimated for biochemical features and grain yield of triticale plants across six years of study

**Normal irrigation**												
	^a^H_2_O_2_	MDH	TChl	Car	Prtn	PRL	POD	CAT	APX	GR	SOD	Yld
genotypic variance	0.05	0.70	2.10	0.83	0.31	1.20	1.03	0.04	21.87	0.70	1.64	41.16
environmental variance	0.20	1.01	5.84	0.51	4.22	5.02	2.90	0.45	19.18	0.73	4.05	173.9
phenotypic variance	0.25	1.71	7.94	1.34	4.53	6.22	3.93	0.49	41.06	1.43	5.69	215.1
heritability	21.8	40.8	26.4	61.9	6.81	19.3	26.1	8.89	53.28	48.7	28.8	19.14
PCV	7.51	42.1	43.8	25.7	31.2	69.8	29.7	23.8	33.43	54.6	47.5	28.99
GCV	1.64	17.2	11.5	15.9	2.12	13.5	7.75	2.12	17.81	26.6	13.7	5.550
mean	3.33	4.05	18.1	5.19	14.5	8.91	13.2	2.07	122.8	2.61	12.0	741.1
**Drought stress**												
	H_2_O_2_	MDH	TChl	Car	Prtn	PRL	POD	CAT	APX	GR	SOD	Yld
genotypic variance	0.27	0.95	6.06	2.05	0.77	6.850	13.42	0.52	38.55	0.93	20.06	47.06
Environmental variance	4.56	3.76	5.26	1.05	17.7	6.780	20.68	1.59	115.3	1.73	12.85	74.83
phenotypic variance	4.83	4.71	11.3	3.10	18.5	13.63	34.10	2.11	153.9	2.66	32.91	121.9
heritability	5.56	20.1	53.5	56.1	4.15	50.25	39.35	24.7	25.05	34.8	60.96	38.61
PCV	62.8	33.6	85.9	51.2	52.8	71.06	78.38	33.6	91.34	39.4	76.16	39.96
GCV	3.49	6.76	46.0	33.9	2.19	35.71	30.85	8.31	22.88	13.7	46.43	15.43
mean	7.69	14.1	13.2	6.06	35.0	19.18	43.51	6.28	168.5	6.75	43.21	305.1

^a^ Hydrogen peroxide: H_2_O_2_, malondialdehyde: MDH, total chlorophyll: TChl, carotenoid: Car, total protein: Prtn, free proline: PRL, peroxidase: POD, catalase: CAT, ascorbic peroxidase: APX, glutathione reductase: GR, superoxide dismutase: SOD, and grain yield: Yld, phenotypic coefficient of variation: PCV, genotypic coefficient of variation: GCV

The average values for all triticale genotypes regarding SOD activity, as the most important variable of the study as a result of the highest heritability and genetic variability in drought stress condition, and grain yield across all 6 years experiments doubled with their standard errors for each stress condition are presented in Table 2. Genotypes 21 (849.5 g/m^2^) and 17 (625.1 g/m^2^) under normal irrigation condition and genotypes 19 (380.6 g/m^2^) and 7 (257 g/m^2^) under drought stress condition showed the highest and the lowest grain yield, respectively. Genotype 3 showed above average grain yield for normal irrigation condition while lower than average point for stress condition; whereas, the grain yield of genotype 28 was above average point under both normal and stress conditions. Genotype 28 showed the highest SOD activity than all other triticale genotypes in both normal irrigation (17.5 u/mg protein) drought stress conditions (49.9 u/mg protein). The lowest SOD activity for normal and stress conditions were obtained in genotype 1 (4.4 u/mg protein) and genotype 3 (16.4 u/mg protein), respectively (Table 2).

**Table 2:** Mean ± standard error of each triticale genotype across six years experiments, separated according to the normal irrigation and drought stress conditions

Genotype	Superoxide dismutase (SOD) activity (u/mg Prt)		Grain yield (g/m^2^)	
	Normal	Stress	Normal	Stress
1	^*a*^*4.40 ± 1.27*	36.6 ± 3.15	669.3 ± 30.00	309.3 ± 16.28
2	5.70 ± 1.27	36.9 ± 3.13	741.5 ± 29.79	323.9 ± 19.16
3	6.70 ± 1.29	*16.4 ± 3.08*	780.4 ± 26.82	264.9 ± 20.53
4	9.30 ± 1.49	39.5 ± 2.94	718.1 ± 33.65	312.5 ± 21.59
5	10.9 ± 1.57	40.0 ± 2.92	772.3 ± 47.61	313.5 ± 25.16
6	11.0 ± 1.59	40.3 ± 2.91	744.3 ± 46.27	320.7 ± 21.61
7	11.2 ± 1.60	20.9 ± 2.93	678.5 ± 35.15	*257.0 ± 17.77*
8	8.72 ± 1.73	21.2 ± 2.92	667.3 ± 29.64	288.2 ± 17.20
9	12.7 ± 1.72	41.7 ± 2.88	825.9 ± 44.12	318.3 ± 20.23
10	10.9 ± 1.76	43.9 ± 2.89	777.6 ± 58.12	360.8 ± 29.89
11	13.1 ± 1.76	42.4 ± 2.81	795.7 ± 34.13	314.6 ± 21.04
12	12.6 ± 1.69	23.3 ± 2.86	845.1 ± 39.87	267.0 ± 12.87
13	12.5 ± 1.63	43.4 ± 2.67	672.6 ± 44.96	337.1 ± 20.40
14	7.81 ± 1.64	43.8 ± 2.68	800.2 ± 33.60	320.4 ± 18.07
15	13.1 ± 1.67	24.5 ± 2.70	776.2 ± 37.10	300.3 ± 13.66
16	13.0 ± 1.65	44.9 ± 2.68	691.4 ± 26.37	318.2 ± 20.86
17	13.7 ± 1.81	35.8 ± 2.19	*625.1 ± 39.74*	261.0 ± 24.98
18	9.90 ± 1.78	45.6 ± 2.14	819.7 ± 44.22	277.2 ± 23.35
19	14.2 ± 1.77	46.5 ± 1.89	745.9 ± 41.58	**380.6 ± 28.93**
20	11.2 ± 2.13	25.2 ± 2.43	761.2 ± 33.29	261.2 ± 13.94
21	7.30 ± 2.19	44.9 ± 2.34	**849.5 ± 45.40**	329.3 ± 13.38
22	11.9 ± 2.19	45.4 ± 2.34	678.2 ± 25.26	290.9 ± 12.91
23	12.0 ± 2.21	34.8 ± 2.15	727.0 ± 48.92	286.5 ± 15.01
24	10.2 ± 2.22	25.4 ± 2.12	756.2 ± 21.64	345.1 ± 13.32
25	13.7 ± 2.11	45.6 ± 2.15	763.7 ± 22.51	313.1 ± 17.91
26	**15.5 ± 1.96**	36.0 ± 2.16	738.5 ± 33.55	301.2 ± 17.93
27	14.0 ± 1.90	46.3 ± 2.16	684.2 ± 25.65	286.1 ± 14.71
28	17.5 ± 2.01	**49.9 ± 1.36**	769.4 ± 26.05	309.8 ± 17.49
29	10.5 ± 1.66	43.3 ± 2.77	727.9 ± 32.12	324.5 ± 22.75
30	12.4 ± 1.68	33.2 ± 2.88	756.9 ± 37.39	269.4 ± 14.40
LSD5%	1.02	2.45	31.22	20.83

^*a*^In each column, **Bolded** and *italicized* number are indicating the highest and lowest mean values, respectively

### Feature selection and promotor analysis

In order to investigate the effects of biochemical features on grain yield of triticale genotypes and finding the most influential features, stepwise regression analysis was performed for normal irrigation and drought stress conditions (Table 3). Accordingly, the results showed that the total protein content was the sole feature selected as the most influential feature on grain yield in normal irrigation condition. The model r-square, known as coefficient of determination, reached to 89% showing a high impact of total protein content on grain yield. Also, the regression coefficient for protein content was negative (b = − 27.79) showing a decreasing effect for this feature toward grain yield under normal irrigation condition. On the other side, stepwise regression model for drought stress condition selected the content of free proline, SOD and GR activities as the influential feature on grain yield of triticale in this condition (Table 3). At the first step of the feature selection for drought stress condition, SOD activity was inserted into model showing that this feature is the most influential independent variable toward the grain yield of triticale. After that, proline and GR activity were respectively inserted into the stepwise model as the second and the third influential variables. The final model of this regression analysis explained about 81% of the total variability in grain yield under the drought stress condition.

**Table 3:** Results of the feature selection based on stepwise regression model regarding the grain yield as response variable for normal irrigation and drought stress conditions

**Normal irrigation**						
Entered Variable		Removed variable	Partial R^2^	Model R^2^	F Value	*p* > F
Protein		^a^NA	0.89	0.89	1395.98	0.017
Over all ANOVA of the final model of selected variables						
Source		DF	Sum of squares	Mean Square	F Value	*p* > F
Model		1	881.368	881.36	52.67	<.0001
Error		538	9002.63	16.73	NA	NA
Parameter estimation for the final model of selected variables						
Variable	Parameter	Standard parameter	Standard error	Sum of squares	F Value	*p* > F
Intercept	1030.13	NA	4.12948	39,289	62,229.6	0.003
Protein	−27.7947	−2.40241	0.74391	881.36	1395.98	0.017
**Drought stress**						
Entered Variable		Removed variable	Partial R^2^	Model R^2^	F Value	*p* > F
SOD		NA	0.520	0.5195	73.02	<.0001
Proline		NA	0.244	0.7635	28.23	<.0001
GR		NA	0.042	0.8055	2.710	0. 003
Over all ANOVA of the final model of selected variables						
Source		DF	Sum of squares	Mean Square	F Value	*p* > F
Model		3	2,380,092.0	793,364	35.99	<.0001
Error		536	11,813,999	22,041.0	NA	NA
Parameter estimation for the final model of selected variables						
Variable	Parameter	Standard parameter	Standard error	Sum of squares	F Value	*p* > F
Intercept	821.479	NA	40.6503	9,001,165	408.4	<.0001
Proline	−4.14588	−0.173164	2.51802	59,751.00	2.710	0.030
SOD	4.65863	0.347539	0.86511	639,160.0	29.00	<.0001
GR	−8.43598	−0.102254	0.84753	2,183,721	99.08	<.0001

^a^ Not available: NA, glutathione reductase: GR, superoxide dismutase: SOD, probability level: p

**Fig. 1:**
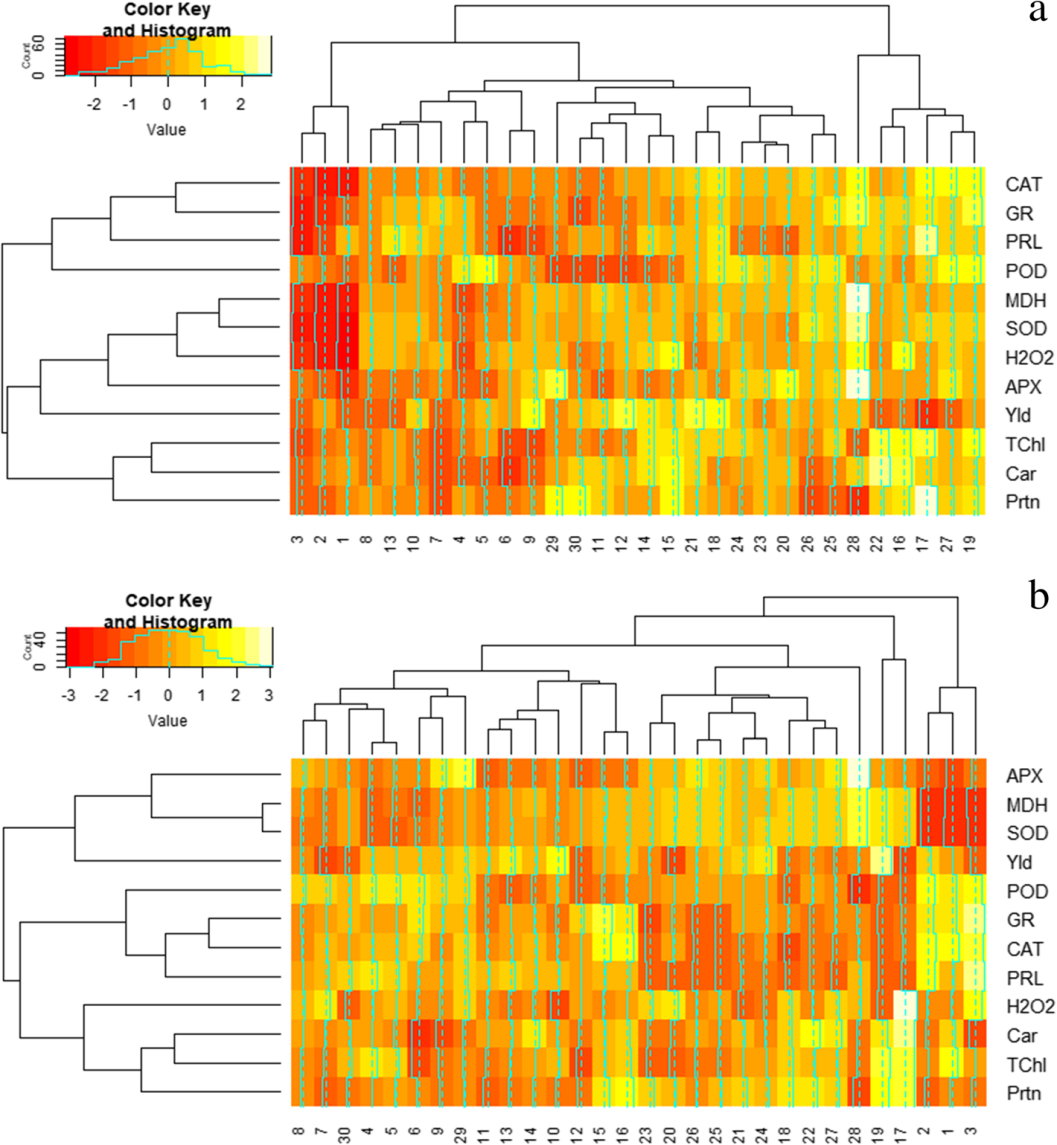
Heatmap showing the saturation of colors indicating the rate and the type of associations between features and triticale genotypes in normal irrigation (**a**) and drought stress (**b**) conditions. Hydrogen peroxide: H_2_O_2_, malondialdehyde: MDH, total chlorophyll: TChl, carotenoid: Car, total protein: Prtn, free proline: PRL, peroxidase: POD, catalase: CAT, ascorbic peroxidase: APX, glutathione reductase: GR, superoxide dismutase: SOD, and grain yield: Yld.

**Fig. 2:**
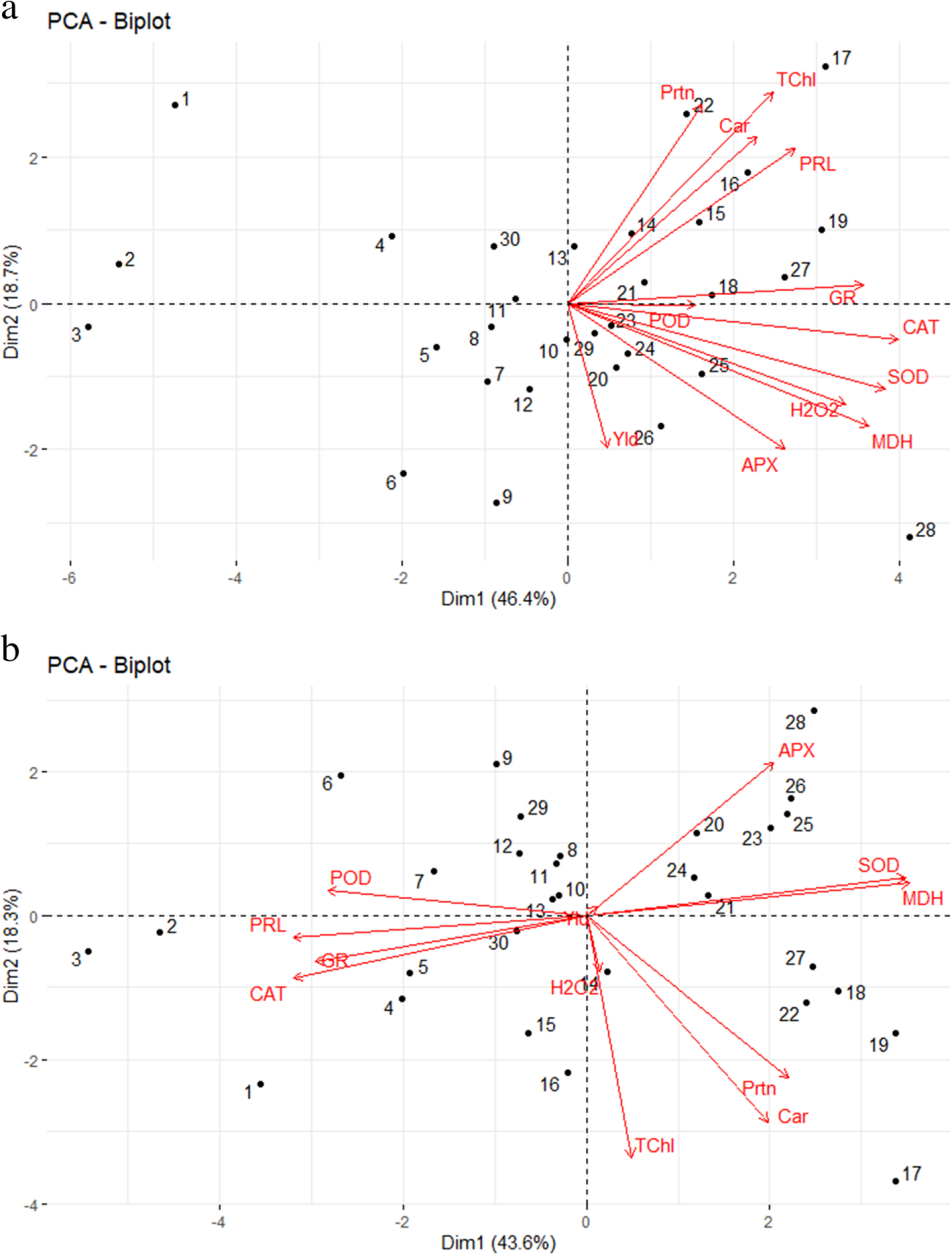
Biplot showing the distribution of the measured feature versus triticale genotypes in two-dimension surface extracted from principal component analysis for normal irrigation (**a**) and drought stress (**b**) conditions. Hydrogen peroxide: H_2_O_2_, malondialdehyde: MDH, total chlorophyll: TChl, carotenoid: Car, total protein: Prtn, free proline: PRL, peroxidase: POD, catalase: CAT, ascorbic peroxidase: APX, glutathione reductase: GR, superoxide dismutase: SOD, and grain yield: Yld

Since the feature selection method based on regression analysis is somewhat biased to availability of any correlation within in depended variables, known as collinearity, a deeper delve over the influential features on grain yield of triticale genotypes was conducted by plotting heatmap, showing the cluster analysis of the measured features under both normal and stress conditions (Fig. 1). Under normal irrigation condition (Fig. 1a), three cluster of the features were pointed out. Subsequently, APX, H_2_O_2_, SOD, and MDH were fallen into the same cluster, while, the protein content fell into a different cluster from the yield. This result verified the result of feature selection and stepwise model, in which the protein content showed a negative slope toward the grain yield. Similarly, SOD, MDH, and APX shared the same cluster with grain yield under drought stress condition (Fig. 1b). Meanwhile, GR and proline, which both showed negative regression coefficients for grain yield in stress condition, were grouped in the same cluster and they were clearly separate from the grain yield cluster. The results of heatmap analysis for drought stress condition corroborated the results of the feature selection model, in which SOD showed a positive influence on the grain yield. Biplot analysis based on the first two components (PC1 and PC2) of principal component analysis (PCA) were applied to find out about the geometrical and structural relationship among measured features and genotypes at the same time. The overall of roughly 64% of the total variation among all features was explained by PC1 and PC2 in normal irrigation condition (Fig. 2a). This two-dimensional (2D) plot showed that, under normal condition, APX, MDH, H_2_O_2_, SOD, POD, and CAT were placed in the same quarter with the grain yield, while the rest of the features were placed in another quarter. Consequently, the arrow of total protein content in normal irrigation condition had the most obtuse (widest) angle with yield arrow indicating negative relationship of this feature as compared with other variables, approving, once again, the result of the feature selection model and heatmap analysis. In a similar pattern to normal irrigation condition, around 61% of total variation among features under drought tress condition was explainable by PC1 and PC2 extracted from PCA. However, the distribution of measured features under drought stress condition was significantly wider than normal irrigation condition; the features were scattered among all four quarter of the biplot (Fig. 2b). Grain yield, MDH, SOD, and APX were distributed in the same quarter of biplot indicating the positive association among these variables. CAT, GR, and proline content were the features in the same quarter directly reverse to the grain yield arrow, showing the negative association of these feature with yield, that once more, verifies the results achieved by feature selection model and the heatmap analysis in drought stress condition.

Among all enzymatic antioxidants considered in this study i.e. SOD, POD, GR, CAT, and POD, all advanced statistical methods selected SOD as the most effective enzyme on grain yield. However, the results achieved by the appropriate statistical methods are clearly indicated by the phenotypic properties and therefore, molecular and genotypic investigations would enable us to test these results. Consequently, promotor analysis of the used enzymatic antioxidants in this study was appraised so as to find any different or similar pattern regarding their regulation mechanism to explain and/or test the actual influence of SOD in drought stress condition. Unfortunately, there is still no bioinformatic database for triticale genome sequence, so we performed the bioinformatic investigation of promotor analysis in wheat genome, its parental line that shares the same AA and BB genomes with triticale. The regulatory elements that were identified in the promotor regions of the enzymatic antioxidants along with their corresponding carrier genome sets are presented in Table 4. The results showed that the greatest number of the enzymatic antioxidant genes were distributed among A and D genome groups. Also, the results revealed a significant difference between the regulatory elements of SOD genes and the regulatory elements of the other antioxidant genes. A high number of regulatory elements in promotor regions of SOD genes were related to circadian regulation, abscisic acid production, auxin response pathways, MYB binding sites, and methyl jasmonate responsive genes that are important compounds of stress responses in plants [4] and were mostly distributed in A chromosome set. In addition, gibberellin and light responding related regulatory elements showed high distributions in SOD promotor regions, especially in A genome set. The total numbers of regulatory elements of SOD promotor regions (1441) were significantly higher than any other considered antioxidant (Table 4).

**Table 4:** Results of the promotor analysis for enzymatic antioxidants in different sets of chromosomes in wheat

Enzyme	Genome set	circadian control	endosperm expression	abscisic acid responsive	auxin-responsive	cell cycle regulation	defense and stress responsive	ethylene-responsive	fungal elicitor	gibberellin-responsive	heat stress responsive	light responsive	low-temperature responsive	MeJA-responsive	meristem specific activation	MYB binding site	salicylic acid responsive	Sum
	A	^a^3	6	1	3	0	0	0	3	5	3	44	1	3	5	2	5	250
GR	B	0	0	0	0	0	0	0	0	0	0	0	0	0	0	0	0	0
	D	1	3	5	1	0	0	1	2	6	0	42	1	3	3	9	3	209
	Sum	4	9	6	4	0	0	1	5	11	3	86	2	6	8	11	8	459
	A	0	2	7	0	1	0	2	3	5	16	21	29	3	2	4	39	286
APX	B	2	0	2	2	0	2	0	0	0	4	15	22	2	2	1	15	141
	D	0	2	1	0	0	0	0	1	3	4	8	9	1	0	2	21	99
	Sum	2	4	10	2	1	2	2	4	8	24	44	60	6	4	7	75	526
	A	1	5	4	0	0	1	0	0	5	1	157	12	0	0	0	13	277
CAT	B	0	0	1	2	0	0	0	0	0	0	79	4	0	4	2	4	137
	D	0	2	2	0	0	1	0	0	1	0	47	4	0	0	3	6	85
	Sum	1	7	7	2	0	2	0	0	6	1	283	20	0	4	5	23	499
	A	4	2	2	0	4	3	2	0	1	1	4	0	1	1	2	4	145
POD	B	0	0	0	4	0	0	0	0	0	0	0	0	0	0	0	0	0
	D	0	1	0	0	5	6	0	0	1	1	2	0	0	5	4	4	205
	Sum	4	3	2	4	9	9	2	0	2	2	6	0	1	6	6	8	350
	A	16	41	30	9	0	0	0	0	9	0	126	0	43	0	41	0	948
SOD	B	0	0	0	0	0	0	0	0	0	0	0	0	0	0	0	0	0
	D	17	5	9	9	0	0	0	0	6	0	69	0	2	0	9	0	476
	Sum	33	46	39	18	0	0	0	0	15	0	195	0	45	0	50	0	1441

^a^The number in each cell shows a number of regulatory elements in the promotor region of the corresponding antioxidant gene regarding one genome set

### Genotype selection for expression analysis

**Fig. 3:**
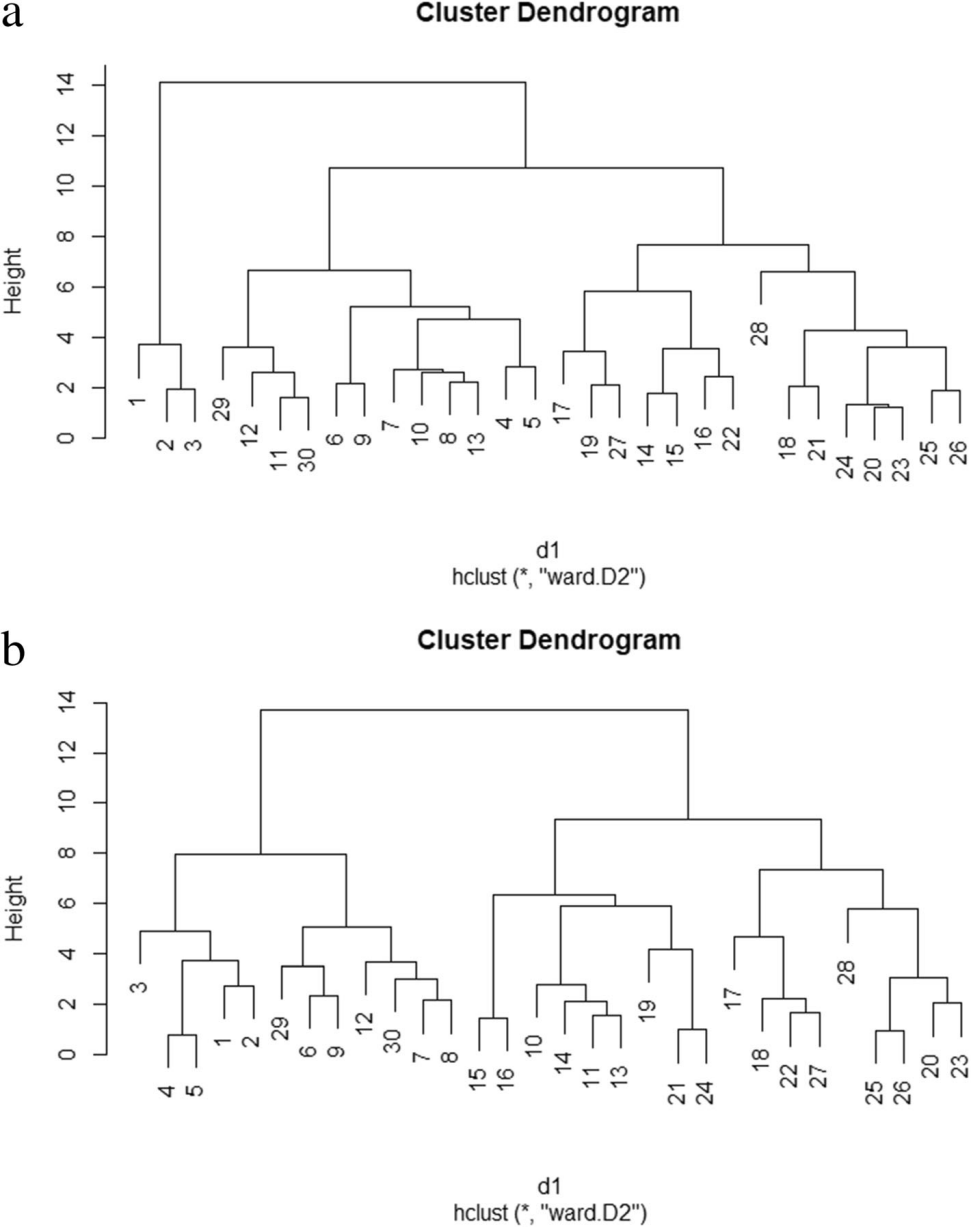
Cluster dendrogram showing the distance between triticale genotypes in normal irrigation (**a**) and drought stress (**b**) conditions

According to the heritability, genotypic variability, using advanced biostatistics methods and different multivariate models alongside bioinformatics approaches, it was clarified that SOD was the most effective antioxidant under drought stress condition in triticale. Therefore, for achieving higher triticale grain yield under drought stress condition, SOD activity has the capability of being used as an indirect criterion, in order to be used in breeding programs of triticale. Therefore, for differential expression analysis goals regarding the genes involved in the SOD response pathways, all of the used triticale genotypes in this study were assessed to find those with the highest genetic distance. Accordingly, the similarities and distances between all pairs of 30 triticale genotypes were considered based on the Euclidean distance method and then, they were transformed into two separate cluster dendrograms, representing both normal and stress conditions (Fig. 3). Under normal irrigation condition, three distinct clusters were distinguished (Fig. 3a). This cluster dendrogram indicated that genotypes 28 and 1 were the two cases presenting the highest genetic dissimilarity, or distance, based on all measured featured. However, genotypes 2 and 3, that were grouped in the same clustered as the genotype 1, were two alternatives for being considered as the genotypes revealing the highest genetic distance from genotype 28, rather than genotype 1. Under drought stress, the highest genetic distance was observed between genotype 28 and genotype 3 (Fig. 3b). Similar to the normal condition result, genotypes 1 and 2 were placed in the same cluster with genotype 3 under drought stress condition. Accordingly, the alternatives for genotype 28 could be genotype 17 and genotype 19 that were in same cluster as the genotype 28. As a result, genotype 1, 2, and 3 versus genotypes 17, 19, and 28 were the genotypes with the highest genetic distances that could be considered in the expressional study. These results were clearly verified by the results obtained from the heatmap analyses (Fig. 1) in which genotype 1, 2, and 3 showed the similar colors for almost all measured features, while they showed very dissimilar colors from genotypes 28, 17, and 19 (Fig. 1a and Fig. 1b). Furthermore, considering the colors saturation in heatmap for these two genotype sets, revealed that they showed the highest dissimilarity regarding the activity of SOD enzyme. However, we were obliged to select just two nominees as the genotypes with the highest dissimilarity for differential expression study. This requirement drew out our attention into biplot and mean comparison for SOD and grain yield. The biplot graphs indicated that, under both normal irrigation and drought stress conditions, genotype 28 was the closest to SOD alongside the grain yield, among the mentioned genotypes (Fig. 2a). Conversely, genotypes 1, 2, and 3 showed similar distance from SOD and grain yield under both normal and stress conditions. As the last option for finding the most proper genotypes with highest genetic variability, mean comparison for SOD and yield were taken into consideration (Table 4). Finally, according to the results of mean comparison for triticale genotypes under both stress conditions, genotype 28 and genotype 3 were chosen to be applied for investigation of SOD isozyme differential expression.

### Differential expression analysis of SOD isozymes

**Fig. 4:**
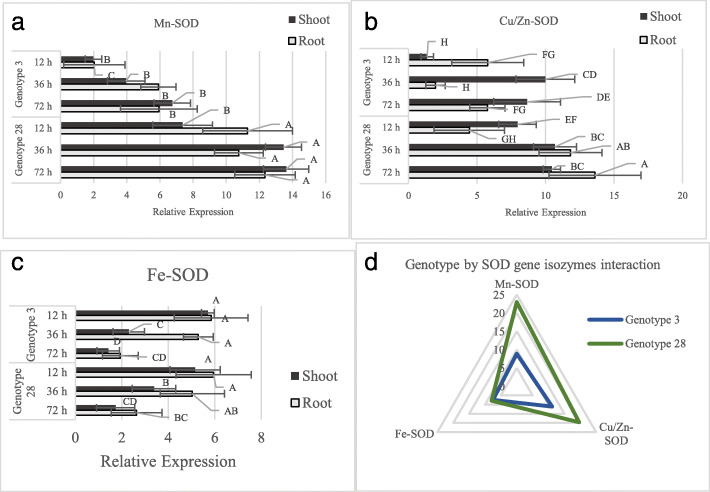
Relative expression average rates of different superoxide dismutase (SOD) isozymes containing Mn-SOD (**a**), Cu/Zn-SOD (**b**), and Fe-SOD (**c**) in two triticale genotypes at different time spans after inducing drought stress along with a net graph showing a comparison relative expression for these isozymes regarding both genotype (**d**). Columns, representing the mean values, with the same letter(s) are not significantly different according to Duncan’s multiple range test (*p* = 0.05).

The relative expression analysis based on real-time PCR method for SOD isozyme containing Mn-SOD, Cu/Zn-SOD, and Fe-SOD was carried on two selected genotypes, genotype 28 and 3, with highest genetic variability with each other. The analysis of variance for the SOD isozymes were carried out based on a factorial experiment, in which genotype and time spans after inducing drought stress were the two experimental factors. The main effect of genotype on Mn-SOD and Cu/Zn-SOD gene expression was significant for both shoot and root tissues, but it did not show a significant effect on Fe-SOD gene expression of these tissues (Fig. 4). Mn-SOD and Cu/Zn-SOD in root tissue were not significantly influenced by the main effect of time after the drought stress, whereas, these two isozymes in the shoot tissue coupled with the Fe-SOD isozyme in both root and shoot tissues showed significant responses to the effect of time spans after drought stress. Except Fe-SOD expression rate in both shoot and root tissues, the two other SOD isozymes showed no significant response to the interaction effect of genotype × time span (Fig. 4).

The Mn-SOD relative expression in both triticale genotypes showed an increasing trend in response to longer duration of time after drought stress in both shoot and root tissues (Fig. 4a). The expression rate of Mn-SOD was higher in shoot than expression rate in the root tissue. Additionally, genotype 28 showed significantly higher expression rate regarding Mn-SOD isozyme in both root and shoot in comparison with genotype 3. The expression rate of Cu/Zn-SOD gene showed different patterns regarding two used triticale genotypes in both shoot and root tissues (Fig. 4b). In genotype 3, Cu/Zn-SOD expression rate in shoot increased by changing the time span from 12 h to 36 h, but there was no significant different between 36 and 72 h in this tissue. Quite the contrary, 36 h showed the lowest Cu/Zn-SOD expression rate than the two other time spans in root tissue of genotype 3. Quite the contrary, the expression rate of Cu/Zn-SOD isozyme in both root and shoot tissues showed a growing (inducing) pattern in response to the longer time span after the drought stress in genotype 28. In both shoot and root tissues of the both genotypes, Fe-SOD expression rate was reduced in response to longer time span, but the decreasing rate in was slower in genotype 28 than the other genotype (Fig. 4c). Comparison the expression rates of the three assessed isozymes of SOD gene with regard to the genotypes, showed significantly higher expression rate of Mn-SOD and Cu/Zn-SOD in genotype 28 than that of genotype 3. The relative expression of Fe-SOD, on the other hand, showed no significant difference between both genotypes.

It should be noted that the measurement of SOD activity in shoot and root tissues showed an increasing pattern in response to longer time span after stress induction in both genotypes. In other words, the longer the drought stress lasted, the higher the activity of SOD enzyme reached. Nevertheless, the SOD activity was significantly higher in genotype 28 in comparison with the genotype 3. Moreover, shoot tissue showed a higher activity of SOD enzyme than that of the root tissue.

## Discussion

In this study, grain yield of triticale and all biochemical features significantly responded to the effects of environment and genotypes as the main sources of variations in the combined ANOVA. This result is indicating the differences among triticale genotypes in response to altered environments, which is alluding to the proficiency of the screening programs for introducing high potential genotypes. Thus, the estimation of genetic parameters would alter in different conditions. Consequently, heritability, PCV, and GCV of almost all features showed significantly higher values in drought stress condition than that in the normal irrigation condition which indicates that screening and breeding programs, in this set of triticale genotypes, would be more efficient in drought stress condition. On the other hand, the main aim of the current study was to find proper methods to reach out to high potential genotype to tolerate drought stress condition. Accordingly, the assessments were done for both normal and stress condition, but the results of the drought stress condition superseded over the results of the normal irrigation condition, if there was any conflict between the results of these two conditions, such as selecting the influential features on grain yield or finding the highest heritable feature. Estimation of genetic parameters for both conditions showed that SOD activity had the highest heritability and GCV under drought stress condition, while it had almost low values of these parameters under normal condition. These results indicate the importance of this enzyme in triticale genotypes under water shortage condition, because GCV shows high variabilities among genotypes and the higher screening ability for finding appropriate genotypes in this regard, also, its high heritability shows high efficiency of screening based on this feature and higher response to selection (RS), than any other feature, in the following generations. In agreement with the result of genetic variability, heatmap analysis clearly showed higher variations of colors among triticale genotypes in relation to SOD activity under drought stress condition. Furthermore, grain yield showed significantly low heritability and genetic variability in comparison with most of other biochemical features, especially SOD activity. Consequently, direct screening for grain yield would not confidently result in higher grain yield in the next generation of breeding program, but using an indirect selection criterion such as SOD for yield would be highly efficient, on condition that it shows a high collinearity and genetical association, either positive or negative, with grain yield. In concordance with heatmap results that showed similar color patterns and saturations for grain yield and SOD activity, and also the result of biplot in which SOD was the closest feature to the grain yield in drought stress condition, the results of the feature selection pointed out that SOD was the most influential feature, with positive impact, on grain yield of triticale. As a result, the capability of SOD for being used as an important indirect criterion for grain yield is verified by all used biometrical and biostatistical methods. To put it simply, genotypes that show higher SOD activity under drought stress condition probably have higher grain yield.

Thus far, there are many studies concerning the effect of environmental stresses on triticale plants, however, few studies have focused on the impacts of biochemical features on grain yield and their capability of being used as indirect criteria; and there is still no study, to the best of our knowledge, considering the heritability and genetic variability of biochemical features in triticale genotypes. In addition, there is still no related program or source code for estimation of genetic parameters e.g. heritability and genetic variability, based on combined ANOVA over different year and location; therefore, the SAS programming code that was used for estimating the biometrical parameters in the current study (supplementary materials) is the first program ever written and practically used to this regard. The results achieved by this code was successfully tested by the results of the hand calculations and the both results were in agreement with each other, except just a little difference as a result of different decimal rounding. It also should be noted that, application of heatmap in the current study and using color saturation for identifying the association between yield and biochemical feature in order for finding possible selection criteria, has never been applied by any other study before. Consequently, our results showed the efficiency of heatmap analysis in screening and breeding programs and also showing a combination of relationships within features, genotypes, and cross relationships between features and genotypes. In accordance with our results regarding the importance of SOD activity, the relationship of biochemical features and grain yield of triticale was modeled in the study of Saed-Moucheshi [45], without estimation of their heritability and other genotypic parameters, and it showed that showed that MDH, proline contents and SOD were the important features with significant impacts on triticale grin yield. Similarly, Riasat et al. [43] nominated SOD activity as the most appropriate trait to obtain higher tolerant triticale genotypes. In addition, there are several studies on different crops that are in concordance with our results regarding the importance of biochemical features as the selection criteria [1, 33, 44, 54, 58]. Saed-Moucheshi et al. [47] showed that SOD along with CAT and proline content were the effective biochemical traits toward the grain yield of barley genotypes and they introduced these traits as the candidate features capable of being used as indirect criteria in barely breeding. However, in the study of Vosough et al. [58], SOD was not able to be selected as an important feature toward grain yield of wheat landraces.

The results of genetic parameters estimation, feature selection, heatmap analysis, and biplot graph alluded to SOD as the most important antioxidant in triticale under drought stress condition. However, these results are based on phenotype and indirect possible products of gene actions, which cannot show the molecular changes inside the cells. Therefore, in order to evaluate the antioxidants at the genomic and molecular level and revealing any plausible similar or dissimilar pattern regarding their regulation mechanisms explaining the aforementioned results, the upstream sequences of the antioxidant genes were appraised by bioinformatics tools. This method through which, the upstream sequences of a gene is evaluated, is known as promotor analysis. The result of promotor analysis showed clear differences between promotor region of SOD and other antioxidant genes. The number of regulatory elements in SOD promotor region was significantly higher than the other antioxidants, mostly by twice as many regulatory elements as others. Also, most of the regulatory elements in SOD promotor region were compounds related to stress responsive pathways. The numerous regulatory elements in promotor region of SOD indicates that this gen can be regulated by various responsive pathways and is most likely more active than other antioxidant in response to changing conditions. In addition, high number of stress related elements in this region shows that this gene is highly responsive to stress condition and it may be highly increased by occurring stress condition. The result also showed a great number of MYB binding sites in the promotor region of SOD genes, that was significantly higher than other antioxidant genes. MYB elements are specifically acting in the responses to water shortage condition, verifying the importance of SOD under drought stress condition.

By combining the results obtained from field study regarding the high heritability and influence of SOD in drought stress condition and the results of promotor analysis showing a high number of stress responsive regulatory elements for this gene, the importance of SOD to increase the tolerance of plants under drought stress condition was clearly verified. SOD is one of the active antioxidant enzymes in dismutation pathways of oxygen free radicals. This enzyme orchestrates the breakdowns of the superoxide radicals (O_2_^−^) into hydrogen peroxide (H_2_O_2_) in the different organelles [20, 29, 46]. Since superoxide radial is the most abundant radicals in some organelles, such as chlorophyll cells [16, 25, 28, 42], it might be said that SOD is the first defense level against occurring oxidative stress in plants [48] which is concordance with the results of the current study regarding high number of stress regulatory elements making SOD to quickly respond to the changing environment. Lin et al. [32] reported that considering the regulatory elements within promotor region of any encoding gene is one of the important strategies to study the molecular responses of plants to different environments. By occurring drought stress in the plant tissues, the plant starts to produce some signal components in accordance with the type of stress, and leads them to reach out to the highly responsive genes [41]. These responsive genes are again producing some other regulatory proteins and compounds and transfer them toward the required genes. These signaling proteins and compounds are known as cis acting regulatory elements [21]. Additionally, most part of the expression regulatory mechanism of plant genes is located within 1000 base pairs of the upstream region to the transcription initiation site [13]. Accordingly, to regulate the expression of SOD genes, a number of cis acting regulatory elements must be present in the promoter region of this gene to regulate its expression based on intracellular changes. Therefore, higher number of regulatory elements that are able to bind with the regulatory motifs in the promotor region bring about higher sensitivity of the corresponding gene to intracellular changes, such as what was identified for SOD promotor in this study. A recent study [24] on the expression pattern of SOD genes in grape wine, showed that its regulatory mechanisms could be active at the three levels of transcription, post-transcription, and translation, and their functional roles are focusing on response to stresses during plant development indicating, in concordance with our results, the high importance of SOD expression under environmental stress conditions and the effect of the regulatory elements in this pathway.

The overall results of biometrical, biostatistical, and bioinformatics analyses indicated that SOD was the most important antioxidant in response to drought stress condition. Also, combining the final results regarding clustering, biplot graph, and heatmap analysis along with the mean comparison for SOD activity and triticale grain yield led to the selection of two genotypes, i.e. genotype 28 and genotype 3, with the highest genetic distance in order to assess the deferential expression profiling of SOD genes in triticale. Considering the activity of SOD enzymes, in the two selected genotypes, showed that the activity of SOD was increased in both genotypes in response to longer time span from initial point of drought stress, but the rate of increase in genotype 28 was over threefold higher than genotype 3. Accordingly, the rate of relative expression of Mn-SOD was about threefold, and Cu/Zn-SOD was about twofold higher in genotype 28 than those in genotype 3. The expression levels of Fe-SOD showed no significant difference between the two genotypes. These results indicated that higher activity of SOD enzyme under drought stress condition is most likely due to the higher expression level of Mn-SOD and/or the Cu/Zn-SOD isozymes, while Fe-SOD is, seemingly, not significantly involved in response to the drought stress. In addition, quite the contrary to Mn-SOD and Cu/Zn-SOD, the expression pattern of Fe-SOD was decreased in response to longer time span from drought stress starting point. This result indicates that Fe-SOD is probably more responsive to non-stress signaling pathways and its expression level under drought stress is decreased so as to let the two other SOD isozymes to act and grow their expression level more rapidly. Furthermore, a comparison between Mn-SOD and Cu/Zn-SOD indicated that the expression level of Mn-SOD is continuously increased as the time span increased in both genotypes; while, the Cu/Zn-SOD showed altered pattern in response to different time spans in each genotype. Therefore, these results are possibly pointing out that Mn-SOD is a general responsive isozyme that its expression level is, somewhat, quite independent of the genetic differences between the genotypes within a species, on the other hand, Cu/Zn-SOD is more dependent on genotypic differences between the two genotypes. In addition, the result related to the expression levels of Cu/Zn-SOD made us to announce it as a genotype specific isozyme, meaning that its expression level is highly correlated with the genetic contents and background of its carrier individual and is likely more expressed in the genotypes tolerant to drought stress. Furthermore, the expression level of Mn-SOD in shoot and root tissues was roughly equal. Contrarily, the expression rate of Cu/Zn-SOD in genotype 3 was higher in shoot than that in root, while its expression level in genotype 28 was significantly lower in shoot than that in the root tissue. The first organelles that are affected by drought stress are normally those in the shoot [15, 35], consequently, this leads the expression levels of Mn-SOD to be significantly higher in shoot. Higher duration of drought stress causes the stress to reach out more to the root system and involve its organelles as well, as a result, the expression level of Cu/Zn-SOD isozyme in the root tissues, mostly in tolerant genotypes, is increased. Conversely, the expression levels of Fe-SOD were continuously higher in root in comparison with the shoot tissue. This result increases the likelihood involvements of Fe-SOD isozyme in non-stressful responsive pathways, because, as mentioned before, the root organelles are less likely affected by negative impacts of drought stress than the shoot organelles.

SOD has been reported to be a key enzyme in regulating intracellular ROS levels and maintaining normal physiological conditions under oxidative stress caused by effect of water shortage condition [30, 36]. The findings of a study performed by Zhao et al. [61] showed that the SOD expression level and malondialdehyde content in rice were significantly correlated with each other, in addition, the activity of SOD expression level in rice cultivars sensitive to heat stress were much lower than the resistant cultivars. Also, in agreement with our results, Mohammadi et al. [38] showed that Cu/Zn-SOD was significantly expressed in common bean plants under drought stress condition, but the rate of its induction in tolerant genotype was significantly higher than the susceptible one. In concordance with our result regarding the importance of Cu/Zn-SOD as a more specific isozyme that its expression levels are most likely changed by genetic contents of its carrier individuals, Xie et al. [60] stated that it is highly feasible that Cu/Zn-SOD play an important role in regulating total SOD activity and ROS detoxification in stressful condition. Moreover, attempts to regulate Cu/Zn-SOD levels in plant chloroplasts through the gene transfer mechanism resulted in transgenic plants with altered expression of the aimed gene. Overexpression of Cu/Zn-SOD genes has been reported to increase stress tolerance in tobacco transgenic plants [55]. In this study, the transgenic tobacco plants had about 30 times higher SOD activity than that in non-transgenic plants. In the study of Sheoran et al. [51], the expression of Mn-SOD gene in C306 and KAW3717 wheat genotypes was significantly increased in response to drought stress conditions; this study significant consistence between expression rate of Mn-SOD and higher overall enzymatic activity of SOD which is in line with the results of the current study related to the higher expression levels of Mn-SOD in drought stress condition. Consequently, some researchers, such as Awan et al. [4] Sheoran et al. [51] and Wang et al. [59], suggested that Mn-SOD is an important agent that plays an important role in response to drought tolerance, however, our results showed that Mn-SOD is possibly a general responsive isozyme that its response to drought stress is similar (or the same) to other stress conditions.

## Conclusion

The overall results indicated the high possibility of screening favorable and potential genotypes to announce as new triticale cultivars as the result of great variabilities among use genotypes in all six years of the study. Additionally, a new programming code written in SAS language was successfully applied to estimate the genetic parameters based on the results of combined ANOVA for the first time since their introduction. The genetic parameters showed a high heritability and genetic variability for SOD activity under drought stress condition. The results of feature selection, heatmap analysis, and biplot clearly indicated that SOD had a significant effect on triticale production in drought stress condition. In this regard, heatmap graph, which is based on the saturation of different colors that shows the association within variables and cases (genotypes) and also their cross relationships, was successfully used for the first time in this study and it showed to be highly appropriate for screening favorable genotypes and processing the features that are proper for indirect selection in breeding programs. According to the results of promotor analysis for antioxidant enzymes, there was a significant difference between SOD and other antioxidants regarding the numbers and the types of motifs and regulatory elements available in their promotor regions. Bioinformatics study showed that the most of regulatory elements in SOD promotor regions were related to stress responsive pathways, especially drought stress as the MYB binding site. Accordingly, two genotypes with the highest genetic distance were selected to consider the differential profiling of three different isozymes of SOD genes in shoot and root organs of the triticale genotypes. The result showed that tolerant genotype (genotype 28) had significantly higher levels of expression for Mn-SOD and Cu/Zn-SOD than susceptible genotype (genotype 3), while the expression level of Fe-SOD was not significant between these two genotypes. Mn-SOD showed higher expression level in shoot than root, but the expression level of Fe-SOD was conversely higher in the root. Whereas, the expression level of Cu/Zn-SOD in tolerant genotype was higher in root while its level in sensitive genotype was lower in root, in comparison with shoot. Finally, it could be stated that Mn-SOD is a general isozyme that responses positively to stress condition in probably most of the plant species with different genetic backgrounds, while, Cu/Zn-SOD is a genotype specific isozyme that is likely more expressed in some genetic content such as the tolerant genotype of triticale in the current study. Furthermore, genotype 28 can be introduced as a favorable genotype for being selected for further assessments regarding its potentials in drought stress condition.

## Methods

This study was consisting of overall three different types of practical research: the first type was consisted of field experiments for considering the phenotypic features, the second one was comprised of a greenhouse experiment for considering expression levels of SOD isozymes in triticale plants, and the third research was containing bioinformatic evaluation and promotor analysis of antioxidant genes.

### Field study

The field study section of this research was consisted of 12 separate trials that were performed in six consecutive growing seasons (from 2014 to 2019) in three different location: Shiraz, Zarghan, and Sanandaj under two different irrigation regimes: normal irrigation and drought stress. The field experiments in Shiraz location were performed during three years (from 2014 to 2016) at the research station of School of Agriculture, Shiraz University, Shiraz, Fars, Iran (52° 46′ E, 29° 50′ N, altitude 1810 m), the field experiment in Zarghan location was carried out during the year 2017 at the research station of Zarghan Researches and Education Center for Agriculture and Natural Resources, Zarghan, Fars, Iran (52.7135° E, 29.7642° N, altitude 1600 m), and the field experiments in Sanandaj location was conducted during the year 2018 and 2019 at the research station of Researches and Education Center for Agriculture and Natural Resources of Kurdistan Province, Sanandaj, Kurdistan, Iran (47.8038° E35.1679° N, altitude 1900 m). The climatic condition of Shiraz, Zarghan, and Sanandaj locations are mild to warm (dry), warm to hot (dry), and mild to cool (wet), respectively, that provided a high variability of environmental conditions for the study. The average precipitation of the corresponding years was 187 mm in 2014 (Shiraz), 143 mm in 2015, 240 mm in 2016, 145 mm in 2017 (Zarghan), 495 mm in 2018 (Sanandaj), and 515 mm in 2019. Also, the physical and chemical properties of the field soil for each experiment is provided in Supplementary Table 1.

In every year, 28 advanced elite genotypes, originated from international CIMMYT center, and two commercial cultivars (Sanabad, and juanilo) of triticale (Supplementary Table 2) were sown in mid-November (10th to 20th) in two separate trials. In each year, two separate sites in the same location were used for two different conditions containing normal irrigation and drought stress conditions. In all experiments, the irrigation schedule was determined according to the time of depleting about 40% of available soil water capacity (SWC). SWC is the difference between wilting point (WP) and field capacity (FC) based on following formula [19]:

$$ SWC\ (L)= FC\ (L)- WP\ (L) $$

SWC: soil water capacity, FC: field capacity, WP: wilting point.

The WP and FC of the soil were accurately measured just the day before sowing date in every year in order to calculate the soil available water capacity. For measuring FC and WP the method of Samarah et al. [49] was applied in which the soil samples were weighed before and after oven-drying at 105 °C for a period of 24 h. Also, for determining the irrigation schedule, the soil samples of 5 fixed experimental units at the depth of 0–40 cm were collected every day, started in the second day after each irrigation, and transferred to the Soil Science Labs and SWC was regularly determined. The drought stress treatment was implemented by withholding irrigation from starting point of heading stage until the crop harvest. In all experiments, either normal irrigation or drought stress trial, 150 kg urea fertilizer and 100 kg triple superphosphate fertilizer were applied just before the sowing of the triticale seeds. All experiments, in both normal irrigation and drought stress trials (12 overall trials), were arranged based on randomized complete block design (RB) with three replications. Each experimental plot (unit) in all experiments was consisted of four rows with 20 cm between rows and 2 m row length (1 m width by 2 m length). At harvest time, two central rows in each plot were used for measuring grain yield.

### Greenhouse study

Following year to the six years of field experiments (2020), a greenhouse experiment was carried out in the greenhouse of Crop Production and Plant Breeding Department of School of Agriculture, Shiraz University, using two selected triticale genotypes consist of genotype 28 and genotype 3, in order to measure the relative expression rate of SOD isozymes (Mn-SOD, Cu/Zn-SOD, and Fe-SOD) under drought stress condition. The triticale seeds (10 seeds per pot) were sown in plastic pots containing 250 g soil. After emergence, the number of plantlets in each pot was reduce to three plantlets. In order to schedule the irrigation times in the greenhouse experiment, RWC and WP of the soil were accurately determined prior to seeds sowing. The content of water for reaching to 100% of FC for plastic pots were measured by weighing the pots (pot + 250 g soil + the content of water for 100% FC). During the experiment, 8 random pots were daily weighed to determine the content water in soil and when, the water content met 80% FC, the pots were irrigated again up to 100% FC. At four leaves stage, water shortage stress was applied by letting the water content of corresponding units meet 60% FC. At this stage, in order to obtain an accurate and precise relative gene expression, the experimental units were divided into two sets: treatment set was consisted of 18 pots (2 genotypes × 3 time-spans × 3 biological repeats) which used for water shortage stress, and control set was consisted of 6 pots (2 genotypes × 3 time-spans) that used as the control for all three replications within each sampling time for each genotype. This division was done due to the destructive manner of the sampling that took place for both shoot (the youngest leaves) and roots of plants in each experimental unit (pot). Sampling for stressed plants were done at 12 h, 36 h, and 72 h after applying the water shortage stress (after reaching 60% FC water content). Sampled tissues were frozen by liquid nitrogen immediately after their separation and transferred to the genetic lab and kept under 80 °C.

### Bioinformatic study

In order to investigate the sequences of the promoters regions of all genes corresponding the antioxidants activities that were measured in this study i.e. superoxide dismutase (SOD), glutathione reductase (GR), catalase (CAT), ascorbic peroxidase (APX), and peroxidase (POD), the complete sequence (CDS) of the mRNA related to these genes in wheat (because the sequence of these genes in triticale have not been identified yet) were downloaded from NCBI site (https://www.ncbi.nlm.nih.gov/nuccore). The identified accession id number for these sequences were consisting of AF387739.1 for APX, X94352.1 for Cat, D85751.1 for GR, AY857755.1 for POD, and JX398977 for SOD. After that, each sequence was uploaded onto PlantEnsemble site (https://plants.ensembl.org/index.html) and blasted against the whole genome of wheat for finding any sequence that shows over 10E-11 similarity score (e-value less than 10–11). Next, 1000 upstream base pair of each hit of PlantEnsemble site were downloaded as promotor regions for aforementioned genes. Finally, each of the downloaded sequences (promotor regions) were reviewed in PlantCare database site (https://bioinformatics.psb.ugent.be/webtools/plantcare/html) for distinguishing any probable regulatory elements. For further investigation and more accurate distinguishing of the regulatory elements, RSAT database (https://rsat.ulb.ac.be/rsat) was used for searching among all sequences for all genes at the same time. All distinguished regulatory elements were then imported into excel files for further investigation and analysis.

### Measurements

In all field experiments of this study (6 years by 2 stress levels), 25 days after drought stress induction, the flag leaves were sampled for measuring biochemical and antioxidant-related features. The flag leaves were immediately frozen by liquid nitrogen and transferred to refrigerators having the temperature below 80 °C till the time of feature measuring. The biochemical features containing content of hydrogen peroxide (H_2_O_2_) [2], content of malondialdehyde (MDH) [22], the amount of total chlorophyll (TChl) [31], free proline (PRL) [5] and total protein (Prtn) [8] contents were measured along with the activities of antioxidant enzymes i.e. glutathione reductase (GR) [52], ascorbic peroxidase (APX) [39], catalase (CAT) [14], peroxidase (POD) [9], and superoxide dismutase (SOD) [6]. The content of H_2_O_2_ and MDH were reported as micro mol per gram (μmol/g) fresh weight (FW), the amount of chlorophyll and protein as milligram per gram (mg/g) FW, and proline content as micromolar per gram (μM/g) FW. The activity of enzymatic antioxidants was reported as micromole of free radicals decomposed in mg of total protein in the sample (u/mg Prt). In addition, at the harvest time, grain yield of the two central rows in each plot were harvested and their grain yield were recorded according to kilogram per square meter (kg/m^2^).

After sampling the youngest leaves of the plants in greenhouse study at each time span, after induction of the drought stress, the activity of SOD enzyme was measured along with the relative expression of SOD isozymes. For extracting the RNA from leaf samples, Dena Zist isolating kits were used. After the extraction, the quantity of extracted RNA was measured by nanodrop method. Following the quantity measurement of RNA, the extracted samples were treated with DNase in order to remove any DNA contamination, afterward, the positive and negative quality control (QC) of the RNA in each sample were performed; the positive QC was checked by direct electrophoresis of 3 mL extracted samples on gel (1% of 0.5 TBE buffer) and observing two separate and sharp bands on the gel representing each of 16 s and 24 s RNA in the samples, while, the negative QC was checked by observing no band on electrophoresis gels posterior to polymerase chain reaction (PCR) of samples conducted by using the primer of internal control (housekeeping: elongation factor 1) gene that was used in this study. Then, the RNA of the extracted samples was transformed into complementary DNA (cDNA) by use of Fermentas Co. kits. The produced cDNA’s were then used for measuring the relative expression of three isozymes of SOD gene i.e. Mn-SOD, Cu/Zn-SOD, and Fe-SOD based on RT-PCR method. The corresponding pair of primers for each isozyme was designed in AlleleID software according to the sequence of the isozymes in wheat genome. In this process, the elongation factor 1 (EF1) was used as the internal control (housekeeping) gene. Each pair of forward (F) and backward (revers: R) primers that were used for expression analysis of Mn-SOD, Cu/Zn-SOD, Fe-SOD, EF1 are as follow:

**Table:** 

Target gene	Primer sequence
Mn-SOD	F: 5′ AACATCTGGAAGGTGGTGAACT 3’
Mn-SOD	R: 5′ AACTCAAGAGCGAGCGAAGTA 3’
Cu/Zn-SOD	F: 5′ CTCCATGAGTTCGGTGACAT 3’
Cu/Zn-SOD	R: 5′ GACGGACTTCATCTTCTGGT 3’
Fe-SOD	F: 5′ GAATTCCACTGGGGAAGCATC 3’
Fe-SOD	R: 5′ GTAAGCGTGCTCCCAAACGTC 3’
Elongation Factor1	F: 5′ CACTGGTCTGACAACTGAGG 3’
Elongation Factor1	R: 5′ GCAACATTCTTGACATTGAAGC 3’

In order to finalize the relative expression measurement, the cycle threshold (Ct) values (automatically calculated by StepOnePlus™ system) of the isozymes and EF1 in each sample, belonging to either treated or control set, were subtracted from each other to obtain ΔCt value (ΔCt = Ct_SOD_ – Ct_EF1_); and then, ΔCt values of samples in treatment set were transformed into relative expression as the proportion of ΔCt values of their control samples (ΔΔCt = ΔCt_trt_ – ΔCt_ctrl_).

### Statistical analysis

The obtained data related to field experiments were subjected to combined analysis of variance (combined ANOVA) and descriptive statistic’s, such as mean and standard error. After that, the mean squares (MS) obtained from combined ANOVA for each of normal irrigation and drought stressed trials were used for estimating the residual (res) variance, variance of block (blk) within year (yr), genotype (gn) by year variance, genotypic variance, environmental variance, phenotypic variance, broad sense (general) heritability, genotypic coefficient of variation (GCV), and phenotypic coefficient of variation (PCV) based on the following formulas [18, 45]. Mean comparison based on LSD test (*p* = 0.05) was carried out using MEANS statement in PROC GLM of SAS software.

**Table:** 

Parameter	Index	Formula
Residual variance	δ^2^_res_	MS_res_
Block (year) variance	δ^2^_blk(yr)_	)MS_blk(yr)_ -MS_res_ (/ (yr × gn)
Genotype by environment variance	δ^2^_gn × yr_	)MS_gn × yr_ -MS_res_ (/ blk
Genotypic variance	δ^2^_gn_	)MS_gn_-MS_res_ (/)blk × yr)
Environmental variance	δ^2^_yr_	)MS_blk(yr)_-MS_gn × yr+_δ^2^_res_ (/ (blk × gn)
Phenotypic variance	δ^2^_pn_	δ^2^_gn_ + [δ^2^_yr_/) blk × yr)] + [δ^2^_gn × yr_ / yr]
General heritability	h^2^	δ^2^_gn_ / δ^2^_pn_
Genotypic coefficient of variation	GCV	[)√δ^2^_gn_(/ Mean] × 100
Phenotypic coefficient of variation	PCV	[)√δ^2^_pn_(/ Mean] × 100

ANOVA and descriptive statistic were analyzed by proc. GLM and proc. MEANS in SAS statistical software version 9.4, respectively. On the other hand, since there is no related program being able to estimate the above-mentioned biometrical and breeding parameters, a code in SAS software was written by the first author to directly calculate these parameters and is provided in the supplementary materials section. In addition, all data generated or analysed during this study are included in this published article as the supplementary information file in Excel (‘xlsx’) format which contains the data for both normal and stress condition in two separate sheets (‘Normal_Irrigation’ and ‘Drought_Stress’).

Factorial ANOVA based on completely randomized design was used for analyzing expression data by using proc. GLM in SAS software. The least significant difference (LSD) method was then used for mean comparison of expression data. Excel software package was used for drawing graphs regarding the mean comparisons alongside evaluating bioinformatics data related to promotor analysis. The mean values of triticale genotype across all years for all measured features were used for building a regression model based on stepwise feature selection method in each of normal irrigation and drought stress conditions. Th stepwise regression model was estimated in SAS software by using proc. REG. Heatmap by use of “gplots” and “agricolae” (for standardizing data) libraries, biplot graph based on principal component analysis (PCA) by use of “factoextra” library, and cluster analysis based on Ward method and Euclidian distance by use of “Nbclust” library were performed in R statistical software (R 3.5).

## Supplementary Information

**Additional file 1: Table S1.** The averaged physical and chemical properties of the soil samples prior to the sowing of the triticale seeds in every year of the study. **Table S2.** Name, id number, and pedigree of the triticale genotypes used in this study. **Table S3.** Combined analysis of variance (ANOVA) for all biochemical features alongside grain yield of triticale. **Table S4.** Analysis of variance for differential expression rate of superoxide dismutase (SOD) isozymes in the root and shoot tissues of triticale genotypes under drought stress condition.

**Additional file 2.**

**Additional file 3.**

## Data Availability

All data generated or analyzed during this study are included in this published article [and its supplementary information files].

## Abbreviations

SOD: Superoxide dismutase
CAT: Catalyze
POD: Peroxidase
GR: Glutathione reductase
APX: Ascorbic peroxidase
H2O2: Hydrogen peroxide
MDH: Malondialdehyde
TChl: Total chlorophyll content
Car: Carotenoid content
Prtn: Total protein content
PRL: Free proline content
yld: Grain yield

## References

[1] Ahmad P, Jaleel CA, Salem MA, Nabi G, Sharma S (2010). Roles of enzymatic and nonenzymatic antioxidants in plants during abiotic stress. Crit Rev Biotechnol.

[2] Alexieva V, Sergiev I, Mapelli S, Karanov E (2001). The effect of drought and ultraviolet radiation on growth and stress markers in pea and wheat. Plant Cell Environ.

[3] Alscher RG, Erturk N, Heath LS (2002). Role of superoxide dismutases (SODs) in controlling oxidative stress in plants. J Exp Bot.

[4] Awan SA, Khan I, Rizwan M, Zhang X, Brestic M, Khan A, El-Sheikh MA, Alyemeni MN, Ali S, Huang L. Exogenous abscisic acid and jasmonic acid restrain polyethylene glycol-induced drought by improving the growth and antioxidative enzyme activities in pearl millet. Physiol Plant. 2020. 10.1111/ppl.13247. 10.1111/ppl.13247 33094486

[5] Bates L, Waldren R, Teare I (1973). Rapid determination of free proline for water-stress studies. Plant Soil.

[6] Beauchamp C, Fridovich I (1971). Superoxide dismutase: improved assays and an assay applicable to acrylamide gels. Anal Biochem.

[7] Behera L, Sharma SS, Samal KC (2020). Role of reactive oxygen species in plant development and its detection assays. Biotica Research Today.

[8] Bradford MM (1976). A rapid and sensitive method for the quantitation of microgram quantities of protein utilizing the principle of protein-dye binding. Anal Biochem.

[9] Chance B, Maehly A (1955). [136] assay of catalases and peroxidases. Methods Enzymol.

[10] Chen Y, Gong B, Xi L, Tang L, Zhu W, Xu L, Zeng J, Wang Y, Fan X, Sha L (2019). Effective introgression of wheat D-genome chromosomes into hexaploid triticale (× *Triticosecale Wittm*.) using trigeneric hybrids. Molecular Breeding.

[11] Cheniany M, Ebrahimzadeh H, Masoudi-nejad A, Vahdati K, Leslie C (2010). Effect of endogenous phenols and some antioxidant enzyme activities on rooting of Persian walnut (*Juglans regia* L.). African J Plant Sci.

[12] Cheniany M, Ebrahimzadeh H, Vahdati K, Preece JE, Masoudinejad A, Mirmasoumi M (2013). Content of different groups of phenolic compounds in microshoots of Juglans regia cultivars and studies on antioxidant activity. Acta Physiol Plant.

[13] Das A, Pramanik K, Sharma R, Gantait S, Banerjee J (2019). In-silico study of biotic and abiotic stress-related transcription factor binding sites in the promoter regions of rice germin-like protein genes. PLoS One.

[14] Dhindsa RS, Plumb-Dhindsa P, Thorpe Ta (1981). Leaf senescence: correlated with increased levels of membrane permeability and lipid peroxidation, and decreased levels of superoxide dismutase and catalase. J Exp Bot.

[15] Du Y, Zhao Q, Chen L, Yao X, Zhang W, Zhang B, Xie F (2020). Effect of drought stress on sugar metabolism in leaves and roots of soybean seedlings. Plant Physiol Biochem.

[16] Dvořák P, Krasylenko Y, Ovečka M, Basheer J, Zapletalová V, Šamaj J, Takáč T. In‐vivo light‐sheet microscopy resolves localisation patterns of FSD1, a superoxide dismutase with function in root development and osmoprotection. Plant, Cell & Environment. 2021. 10.1111/pce.13894. 10.1111/pce.13894 32974958

[17] Elshafei AM (2020). When oxygen can be toxic? A mini review. Journal of Applied Life Sciences International.

[18] Fan Z (2018). Eigenvalues in multivariate random effects models.

[19] Givi J, Prasher SO, Patel RM (2004). Evaluation of pedotransfer functions in predicting the soil water contents at field capacity and wilting point. Agric Water Manag.

[20] Hasanuzzaman M, Bhuyan MHM, Zulfiqar F, Raza A, Mohsin SM, Mahmud JA, Fujita M, Fotopoulos V (2020). Reactive oxygen species and antioxidant defense in plants under abiotic stress: revisiting the crucial role of a universal defense regulator. Antioxidants.

[21] Ho C-L, Geisler M (2019). Genome-wide computational identification of biologically significant cis-regulatory elements and associated transcription factors from rice. Plants.

[22] Hodges DM, DeLong JM, Forney CF, Prange RK (1999). Improving the thiobarbituric acid-reactive-substances assay for estimating lipid peroxidation in plant tissues containing anthocyanin and other interfering compounds. Planta.

[23] Hossain MA, Bhattacharjee S, Armin S-M, Qian P, Li H-Y, Burritt DJ, Fujita M, Tran L-SP (2015). Hydrogen peroxide priming modulates abiotic oxidative stress tolerance: insights from ROS detoxification and scavenging. Front Plant Sci.

[24] Lotfi N, Vahdati K, Kholdebarin B, Amiri R. Soluble sugars and proline accumulation play a role as effective indices for drought tolerance screening in Persian walnut (Juglans regia L.) during germination. Fruits. 2009;65:97–112.

[25] Iqbal N, Hussain S, Raza MA, Yang C, Safdar ME, Brestic M, Aziz A, Hayyat MS, Asghar MA, Yang W (2019). Drought tolerance of soybean (*Glycine max* L. Merr.) by improved photosynthetic characteristics and an efficient antioxidant enzyme system under a split-root system. Front Physiol.

[26] Jariteh M, Ebrahimzadeh H, Niknam V, Mirmasoumi M, Vahdati K (2015). Developmental changes of protein, proline and some antioxidant enzymes activities in somatic and zygotic embryos of Persian walnut (*Juglans regia* L.). Plant Cell Tissue and Organ Culture (PCTOC).

[27] Jariteh M, Ebrahimzadeh H, Niknam V, Vahdati K, Amiri R (2011). Antioxidant enzymes activities during secondary somatic embryogenesis in Persian walnut (*Juglans regia* L.). Afr J Biotechnol.

[28] Khodadadi F, Tohidfar M, Mohayeji M, Dandekar AM, Leslie CA, Kluepfel DA, Butterfield T, Vahdati K (2016). Induction of polyphenol oxidase in walnut and its relationship to the pathogenic response to bacterial blight. J Am Soc Hortic Sci.

[29] Khodadadi F, Tohidfar M, Vahdati K, Dandekar AM, Leslie CA (2020). Functional analysis of walnut polyphenol oxidase gene (JrPPO1) in transgenic tobacco plants and PPO induction in response to walnut bacterial blight. Plant Pathol.

[30] Kohli SK, Khanna K, Bhardwaj R, AbdAllah EF, Ahmad P, Corpas FJ (2019). Assessment of subcellular ROS and NO metabolism in higher plants: multifunctional signaling molecules. Antioxidants.

[31] Lichtenthaler HK, Buschmann C (2001). Chlorophylls and carotenoids: measurement and characterization by UV-VIS spectroscopy. Current Protocols in Food Analytical Chemistry UNIT.

[32] Lin K-H, Sei S-C, Su Y-H, Chiang C-M (2019). Overexpression of the Arabidopsis and winter squash superoxide dismutase genes enhances chilling tolerance via ABA-sensitive transcriptional regulation in transgenic Arabidopsis. Plant Signal Behav.

[33] Lotfi N, Vahdati K, Amiri R, Kholdebarin B (2010). Drought-induced accumulation of sugars and proline in radicle and plumule of tolerant walnut varieties during germination phase. VI International Walnut Symposium.

[34] Lotfi N, Vahdati K, Hassani D, Kholdebarin B, Amiri R (2010). Peroxidase, guaiacol peroxidase and ascorbate peroxidase activity accumulation in leaves and roots of walnut trees in response to drought stress. VI International Walnut Symposium.

[35] Lotfi N, Vahdati K, Kholdebarin B, Amiri R (2009). Soluble sugars and proline accumulation play a role as effective indices for drought tolerance screening in Persian walnut (*Juglans regia* L.) during germination. Fruits.

[36] Mao H, Chen M, Su Y, Wu N, Yuan M, Yuan S, Brestic M, Zivcak M, Zhang H, Chen Y (2018). Comparison on photosynthesis and antioxidant defense Systems in Wheat with different Ploidy levels and Octoploid Triticale. Int J Mol Sci.

[37] Mergoum M, Sapkota S, ElDoliefy AEA, Naraghi SM, Pirseyedi S, Alamri MS, AbuHammad W. Triticale (x Triticosecale Wittmack) Breeding, Advances in Plant Breeding Strategies: Cereals. J Plant Res. 2019;405–51.

[38] Mohammadi M, Tavakoli A, Pouryousef M, Fard EM (2020). Study the effect of 24-epibrassinolide application on the cu/Zn-SOD expression and tolerance to drought stress in common bean. Physiol Mol Biol Plants.

[39] Nakano Y, Asada K (1981). Hydrogen peroxide is scavenged by ascorbate-specific peroxidase in spinach chloroplasts. Plant Cell Physiol.

[40] Pirasteh-Anosheh H, Saed-Moucheshi A, Pakniyat H, Pessarakli M. Stomatal responses to drought stress. Water stress and crop plants. 2016:24–40. 10.1002/9781119054450.ch3.

[41] Rasel M, Tahjib-Ul-Arif M, Hossain MA, Hassan L, Farzana S, Brestic M. Screening of salt-tolerant rice landraces by seedling stage phenotyping and dissecting biochemical determinants of tolerance mechanism. J.Plant Growth Regul. 2020. 10.1007/s00344-020-10235-9.

[42] Rezaei Qusheh Bolagh F, Solouki A, Tohidfar M, Zare Mehrjerdi M, Izadi-Darbandi A, Vahdati K (2020). Agrobacterium-mediated transformation of Persian walnut using BADH gene for salt and drought tolerance. J Hortic Sci Biotechnol.

[43] Riasat M, Kiani S, Saed-Mouchehsi A, Pessarakli M (2019). Oxidant related biochemical traits are significant indices in triticale grain yield under drought stress condition. J Plant Nutr.

[44] Riasat M, Saed-Mouchehsi A, Jafari AA (2020). Effect of drought stress levels on seedling Morpho-physiological traits of alfalfa (*Medicago sativa*) populations grown in glasshouse. J Rangeland Science.

[45] Saed-Moucheshi A (2018). Evaluation of morphological, physiological, and moleculare characteristics of triticale genotypes under drought stress condition.

[46] Saed-Moucheshi A, Pakniyat H, Pirasteh-Anosheh H, Azooz M (2014). Role of ROS as signaling molecules in plants.

[47] Saed-Moucheshi A, Pessarakli M, Mikhak A, Ostovar P, Ahamadi-Niaz A (2017). Investigative approaches associated with plausible chemical and biochemical markers for screening wheat genotypes under salinity stress. J Plant Nutr.

[48] Saed-Moucheshi A, Shekoofa A, Pessarakli M (2014). Reactive oxygen species (ROS) generation and detoxifying in plants. J Plant Nutr.

[49] Samarah NH, Alqudah AM, Amayreh JA, McAndrews GM (2009). The effect of late-terminal drought stress on yield components of four barley cultivars. J Agron Crop Sci.

[50] Shakeri E, Mozafari AA, Sohrabi F, Saed-Moucheshi A (2019). Role of Proline and other Osmoregulatory compounds in plant responses to abiotic stresses, handbook of plant and crop stress, fourth edition. CRC press.

[51] Sheoran S, Thakur V, Narwal S, Turan R, Mamrutha H, Singh V, Tiwari V, Sharma I (2018). Differential activity and expression profile of antioxidant enzymes and physiological changes in wheat (Triticum aestivum L.) under drought. Appl Biochem Biotechnol Agronomy Soc.

[52] Smith IK, Vierheller TL, Thorne CA (1988). Assay of glutathione reductase in crude tissue homogenates using 5, 5′-dithiobis (2-nitrobenzoic acid). Anal Biochem.

[53] Stepochkin PI (2019). Study of 8x and 6x triticale with dominant Vrn genes. Current Challenges in Plant Genetics, Genomics, Bioinformatics, and Biotechnology.

[54] Tabarzad A, Ayoubi B, Riasat M, Saed-Moucheshi A, Pessarakli M (2017). Perusing biochemical antioxidant enzymes as selection criteria under drought stress in wheat varieties. J Plant Nutr.

[55] Tepperman JM, Dunsmuir P (1990). Transformed plants with elevated levels of chloroplastic SOD are not more resistant to superoxide toxicity. Plant Mol Biol.

[56] Vahdati K, Lotfi N (2013). Abiotic stress tolerance in plants with emphasizing on drought and salinity stresses in walnut. Abiotic Stress–Plant Responses and Applications in Agriculture.

[57] Vahdati K, Lotfi N, Kholdebarin B, Hassani D, Amiri R, Mozaffari MR, Leslie C (2009). Screening for drought-tolerant genotypes of Persian walnuts (*Juglans regia* L.) during seed germination. HortScience.

[58] Vosough A, Ghouchani R, Saed-Moucheshi A (2019). Genotypic variation and heritability of antioxidant related traits in wheat landraces of Iran. Biological Forum.

[59] Wang W, Xia M, Chen J, Yuan R, Deng F, Shen F (2018). Gene expression characteristics and regulation mechanisms of superoxide dismutase and its physiological roles in plants under stress. Biochemistry.

[60] Xie X, He Z, Chen N, Tang Z, Wang Q, Cai Y (2019). The roles of environmental factors in regulation of oxidative stress in plant. Biomed Res Int.

[61] Zhao Q, Zhou L, Liu J, Du X, Huang F, Pan G, Cheng F (2018). Relationship of ROS accumulation and superoxide dismutase isozymes in developing anther with floret fertility of rice under heat stress. Plant Physiology Biochemistry.

