# Supplementary material for: Superoxide dismutase (SOD) as a selection criterion for triticale grain yield under drought stress: a comprehensive study on genomics and expression profiling, bioinformatics, heritability, and phenotypic variability
Source: BMC Plant Biol. 2021 Mar 22;21:148. doi: 10.1186/s12870-021-02919-5 (PMC7986280; doi:10.1186/s12870-021-02919-5)
Supplement: Supplementary file 1 — Additional file 1: Table S1. The averaged physical and chemical properties of the soil samples prior to the sowing of the triticale seeds in every year of the study. Table S2. Name, id number, and pedigree of the triticale genotypes used in this study. Table S3. Combined analysis of variance (ANOVA) for all biochemical features alongside grain yield of triticale. Table S4. Analysis of variance for differential expression rate of superoxide dismutase (SOD) isozymes in the root and shoot tissues of triticale genotypes under drought stress condition. [file 12870_2021_2919_MOESM1_ESM.docx]

| Supplementary Table 1. The averaged physical and chemical properties of the soil samples prior to the sowing of the triticale seeds in every year of the study. | | | | | | | | | | |
| --- | --- | --- | --- | --- | --- | --- | --- | --- | --- | --- |
| Year | Mn | Cd | Zn | Cu | Fe | K | P | Ec | pH | Texture |
|  | (mg kg^-1^) | (mg kg^-1^) | (mg kg^-1^) | (mg kg^-1^) | (mg kg^-1^) | (mg kg^-1^) | (mg kg^-1^) | (dS m^-1^) |  |  |
| 2014 | 1.4 | 0.3 | 0.60 | 0.40 | 2.5 | 207 | 10.7 | 2.4 | 7.8 | Clay-loamy |
| 2015 | 1.5 | 0.4 | 0.45 | 0.40 | 2.4 | 200 | 10.5 | 2.5 | 7.7 | Clay-loamy |
| 2016 | 1.3 | 0.2 | 0.55 | 0.44 | 2.6 | 208 | 10.8 | 2.4 | 7.8 | Clay-loamy |
| 2017 | 1.0 | 0.2 | 0.53 | 0.48 | 2.4 | 209 | 11.1 | 2.8 | 7.6 | Clay-loamy |
| 2018 | 1.1 | 0.2 | 0.55 | 0.50 | 2 | 210 | 12.8 | 2.5 | 7.0 | Clay-loamy |
| 2019 | 1.2 | 0.2 | 0.49 | 0.51 | 2.2 | 212 | 12.1 | 2.5 | 6.8 | Clay-loamy |

| Supplementary Table 2. Name, id number, and pedigree of the triticale genotypes used in this study. | | |
| --- | --- | --- |
| No. | Genotype | Pedigree |
| 1 | ELTTCL1 | LIRON_2.5.DISB5.3.SPHD.PVN.YOGUI_6.4.KER_3.6.BULL_10.MANATI_1.7.ARDI_1.TOPO1419.ERIZO_9.3.2*KETTU_1 |
| 2 | ELTTCL2 | AR.SNP6.TARASCA87_3.C,S10.3.URON_5.TATU_1.4.BULL_10.MANATI_1.3.ELK54.BUF_2.NIMIR_3.5.DAHBI_6.3.ARDI_1.TOPO1419.ERIZO_9 |
| 3 | ELTTCL4 | BW32_1.CENT.SARDEV.7.LIRON_2.5.DISB5.3.SPHD.PVN.YOGUI_6.4.KER_3.6.BULL_10.MANATI_1.8.MERINO.JLO.REH.3.HARE_267.4.ARDI_4.5.PTR.CSTO.GLT.3.RHINO_4-1.4.HARE_7265.YOGUI_3.6.BULL_10.MANATI_1 |
| 4 | ELTTCL7 | DRIRA.2*CMH77A.1165.8.NIMIR_3.ERIZO_12.5.GC.3.733.EB.MPE.3.LAMB_3.4.BUF_2.6.POLLMER_2.7.FAHAD_8-2.9.ARDI_1.TOPO1419.ERIZO_9.3.LIRON_1-1.4.FAHAD_4.FARAS_1 |
| 5 | ELTTCL8 | CMH80.1212.CMH81A.1239.3.YOGUI_3.ERIZO_11.ONA_2.POSS_1-2.7.LIRON_2.5.DISB5.3.SPHD.PVN.YOGUI_6.4.KER_3.6.BULL_10.MANATI_1 |
| 6 | ELTTCL9 | CMH82.1082.ZEBRA31.7.LIRON_2.5.DISB5.3.SPHD.PVN.YOGUI_6.4.KER_3.6.BULL_10.MANATI_1.8.LIRON_2.5.DISB5.3.SPHD.PVN.YOGUI_6.4.KER_3.6.BULL_10.MANATI_1 |
| 7 | ELTTCL10 | FD-693.2*FAHAD_4.POLLMER_4.3.POLLMER_2.1.4.FARAS.CMH84.4414.6.RHINO_3.BULL_1-1.5.CMH77.1135.CMH77A.1165.2*YOGUI_1.3.IBEX.4.JLO97.CIVET |
| 8 | ELTTCL12 | LIRON_2.5.DISB5.3.SPHD.PVN.YOGUI_6.4.KER_3.6.BULL_10.MANATI_1.7.DAHBI_6.3.ARDI_1.TOPO1419.ERIZO_9 |
| 9 | ELTTCL15 | ARDI_1.TOPO1419.ERIZO_9.3.LIRON_1-1.4.FAHAD_4.FARAS_1.5.DAHBI.3.FAHAD-2-8*2.PTR.PND-T |
| 10 | ELTTCL18 | HX87-244.HX87-255.3.T1502_WG.MOLOC_4.RHINO_3.BULL_1-1 |
| 11 | ELTTCL19 | HX87-244.HX87-255.5.PRESTO.2*TESMO_1.MUSX603.4.ARDI_1.TOPO1419.ERIZO_9.3.SUSI_2 |
| 12 | ELTTCL20 | POPP1_2.TX93-57-7.7.LIRON_2.5.DISB5.3.SPHD.PVN.YOGUI_6.4.KER_3.6.BULL_10.MANATI_1 |
| 13 | ELTTCL21 | TAHARA.TREAT.7.LIRON_2.5.DISB5.3.SPHD.PVN.YOGUI_6.4.KER_3.6.BULL_10.MANATI_1 |
| 14 | ELTTCL22 | POLLMER_2.2.1*2.FARAS.CMH84.4414.4.DAHBI_6.3.ARDI_1.TOPO1419.ERIZO_9 |
| 15 | ELTTCL24 | LIRON_2.5.DISB5.3.SPHD.PVN.YOGUI_6.4.KER_3.6.BULL_10.MANATI_1.7.RHINO_3.BULL_1-1.8.BAT*2.BCN.CAAL.3.ERIZO_7.BAGAL_2.FARAS_1 |
| 16 | ELTTCL25 | PRESTO.2*TESMO_1.MUSX603.4.ARDI_1.TOPO1419.ERIZO_9.3.SUSI_2.5.POPP1_1.6.BULL_10.MANATI_1*2.FARAS.CMH84.4414 |
| 17 | ELTTCL28 | LIRON_2.5.DISB5.3.PHD.PVN.YOGUI_6.4.KER_3.6.BULL_10.MANATI_1*2.7.TUKURU |
| 18 | ELTTCL29 | LIRON_2.5.DISB5.3.SPHD.PVN.YOGUI_6.4.KER_3.6.BULL_10.MANATI_1*2.7.TUKURU |
| 19 | ELTTCL30 | LIRON_2.5.DISB5.3.SPHD.PVN.YOGUI_6.4.KER_3.6.BULL_10.MANATI_1*2.7.TUKURU |
| 20 | ET-90-3 | DAHBI_6/3/ARDI_1/TOPO1419//ERIZO_9/4/SONNI_3 |
| 21 | ET-90-4 | BAT*2/BCN//CAAL/3/ERIZO_7/BAGAL_2//FARAS_1 |
| 22 | ET-90-5 | ARDI_1/TOPO1419//ERIZO_9/3/LIRON_1-1/4/FAHAD_4/FARAS_1/5/CT775.81/ARDI_1//ANOAS_1 |
| 23 | ET-90-6 | DAHBI_6/3/ARDI_1/TOPO1419//ERIZO_9/5/804/BAT/3/MUSX/LYNX//STIER_12-3/4/VARSA_3-1 |
| 24 | ET-90-7 | DAHBI_6/3/ARDI_1/TOPO1419//ERIZO_9/4/NIMIR_1/HARE_265//ERIZO_9/5/RHINO1RS.1DL3384/2*VICUNA_4 |
| 25 | ET-90-8 | POLLMER_2.2.1//FARAS/CMH84.4414/4/LAD622.81/PORSAS_4-1/3/ARDI_1/TOPO1419//ERIZO_9 |
| 26 | ET-90-9 | PRESTO//2*TESMO_1/MUSX603/4/ARDI_1/TOPO1419//ERIZO_9/3/SUSI_2/5/AR/SNP6//TARASCA87_2/C,S10/3/PORSAS |
| 27 | ET-90-10 | PRESTO//2*TESMO_1/MUSX603/4/ARDI_1/TOPO1419//ERIZO_9/3/SUSI_2/5/AR/SNP6//TARASCA87_2/C,S10/3/PORSAS |
| 28 | ET-90-11 | DAHBI/3/FAHAD_8-2*2//PTR/PND-T/7/LIRON_2/5/DISB5/3/SPHD/PVN//YOGUI_6/4/KER_3/6/BULL_10/MANATI_1 |
| 29 | Sanabad | Commercial cultivar |
| 30 | juanilo | Commercial cultivar |

| Supplementary Table 3. Combined analysis of variance (ANOVA) for all biochemical features alongside grain yield of triticale. | | | | | | | | | | | | | |
| --- | --- | --- | --- | --- | --- | --- | --- | --- | --- | --- | --- | --- | --- |
| Source of variation | Degree of freedom | Mean squares | | | | | | | | | | | |
|  |  | ^Λ^H_2_O_2_ | MDH | TChl | Car | Prtn | PRL | POD | CAT | APX | GR | SOD | Yld |
| Stress (S) | 1 | 1128.35** | 7522.55** | 2154.13** | 232.75** | 28466.65** | 7405.94** | 43001.35** | 1047.19** | 129822.26* | 1010.01** | 67829.59** | 14247039.99** |
| Year (Y) | 5 | 333.72** | 3343.56** | 1558.56** | 358.18** | 8372.48* | 1955.15** | 81143.12** | 1160.42** | 119243.14* | 1155.48** | 43911.91** | 572514.98** |
| S*Y | 5 | 421.49** | 2357.07* | 101.26ns | 1201.84** | 178.76ns | 13311.27** | 3248.59ns | 390.28* | 1527.17ns | 389.91* | 8281.53* | 51016.33** |
| Block/L*y | 24 | 14.37 | 2357.07 | 64.83 | 13.13 | 372.89 | 369.87 | 1180.24 | 53.91 | 27143.3 | 61.18 | 1890.04 | 9014.4 |
| Genotype (G) | 29 | 355.78** | 2433.33** | 124.18** | 222.14** | 13421.03** | 1810.46** | 14009.98** | 411.69** | 4447.63** | 809.95** | 12266.51** | 51237.51** |
| S*G | 29 | 143.95** | 902.85** | 23.22** | 10.48** | 716.55** | 1012.08ns | 372.39ns | 151.53** | 4246.21** | 11.83** | 1223.3** | 20026.6** |
| Y*G | 145 | 114.68** | 700.38** | 64.92** | 13.39** | 928.42** | 48.12ns | 994.33** | 161.68** | 2573.14** | 17.7** | 910.63** | 21656.04** |
| S*Y*G | 145 | 33.42** | 111.07** | 3.63** | 10.87** | 92.78* | 1224.95** | 602.4** | 100.15** | 3382.85** | 47.23** | 987.67** | 15234.62** |
| Residual (error) | 696 | 7.33 | 23.34 | 8.88 | 2.81 | 83.23 | 58.44 | 306.43 | 5.25 | 1382.5 | 5.36 | 190.02 | 4027.27 |
| **, * and “ns”: significant at p<0.01, p<0.05 and not significant, respectively.  ^Λ^ Hydrogen peroxide: H_2_O_2_, malondialdehyde: MDH, total chlorophyll: TChl, carotenoid: Car, total protein: Prtn, free proline: PRL, peroxidase: POD, catalase: CAT, ascorbic peroxidase: APX, glutathione reductase: GR, superoxide dismutase: SOD, and grain yield: Yld. | | | | | | | | | | | | | |

| Supplementary Table 4. Analysis of variance for differential expression rate of superoxide dismutase (SOD) isozymes in the root and shoot tissues of triticale genotypes under drought stress condition. | | | | | | | | |
| --- | --- | --- | --- | --- | --- | --- | --- | --- |
| Source | Degree of freedom | Shoot (mean squares) | | |  | Root (mean squares) | | |
|  |  | Mn-SOD | Cu/Zn-SOD | Fe-SOD |  | Mn-SOD | Cu/Zn-SOD | Fe-SOD |
| Genotype (G) | 1 | 236.6** | 41.4* | 19.2ns |  | 210.1** | 133.9** | 25.1ns |
| Time (T) | 2 | 48.6** | 56.8** | 51.4** |  | 9.5ns | 31.8ns | 79.8** |
| G×T | 2 | 6.5ns | 14.7ns | 64.7** |  | 7.4ns | 53.5ns | 71.2** |
| Residual (error) | 12 | 4.5 | 7.7 | 4.1 |  | 11.3 | 16.16429 | 4.51 |
| **, * and “ns”: significant at p<0.01, p<0.05 and not significant, respectively. | | | | | | | | |
