# Supplementary material for: Superoxide dismutase (SOD) as a selection criterion for triticale grain yield under drought stress: a comprehensive study on genomics and expression profiling, bioinformatics, heritability, and phenotypic variability
Source: BMC Plant Biol. 2021 Mar 22;21:148. doi: 10.1186/s12870-021-02919-5 (PMC7986280; doi:10.1186/s12870-021-02919-5)
Supplement: Supplementary file 2 — Additional file 2. [file 12870_2021_2919_MOESM2_ESM.docx]

Supplementary materials

This is the programming code wrote based on SAS language using PROC IML (Interactive Matrix Language) for calculating biometrical genetics parameters that are applied in plant breeding programs in multi environments studies.

/*This code is originally written by Dr. Armin Saed-Moucheshi: saedmoocheshi@gmail.com */

/*This code uses the data from multi locations or multi years studies and estimates biometrical

genetics parameters consisting of: genotypic variance, environmental variance, phenotypic variance,

general heritability, genotypic coefficient of variation, phenotypic coefficient of variation,

response to selection, and next generation means. */

/*The code has successfully been tested in SAS 9.2, SAS 9.3, and SAS 9.4M5*/

%let mod=Env rep(Env) Gen Env*Gen;

%let CLASS= Env Gen Rep;

**Data** n;

input Env $ Gen $ Rep x1-x12;

cards;

;

**proc** **glm** data=n;

class &CLASS;

model X1-X12=&mod/ss3;

manova h=_all_/printe printh;

ods output overallanova=over modelanova=model ErrorSSCP=ESSCP ypothesisSSCP=HSSCP;

**RUN**;

/*PROC PRINT DATA= OVER ; PROC PRINT DATA= MODEL ;

PROC PRINT DATA= ESSCP ; PROC PRINT DATA= HSSCP ; */

**RUN**;

**PROC** **IML**;

USE n (DROP = &CLASS);

READ ALL INTO DATA;

Nrow = Nrow(data);

Ncol = Ncol(data);

Mean = data[+,] / Nrow;

use over; read all into over;

DFE = Over [**2**, **1**];

use model;read all into model;

DFEnv = model [**1**, **2**]; Env = DFEnv + **1**;

DFRepEnv = model [**2**, **2**]; Rep = (DFRepEnv / Env) + **1**;

DFGen = model [**3**, **2**]; Gen = DFGen + **1**;

DFGenEnv = model [**4**, **2**];

use ESSCP; read all into SSE;

MSE = SSE / DFE;

use HSSCP; read all into SSH;

N11 = Ncol + **1**;

N2 = **2** * Ncol;

N21 = N2 + **1**; N3 = **3** * Ncol;

N31 = N3 + **1**;

N4 = **4** * Ncol;

N41 = N4 + **1**; N5 = **5** * ncol;

N51 = N5 + **1**;

SSEnv = SSH [**1** : ncol, **1** : Ncol];

SSRepEnv = SSH [N11 : N2, **1** : Ncol];

SSGen = SSH [N21 : N3, **1** : Ncol];

SSGenEnv = SSH [N31 : N4, **1** : Ncol];

MSEnv = SSEnv / DFEnv ;

MSRepEnv = SSRepEnv / DFRepEnv;

MSGen = SSGen / DFGen ;

MSGenEnv = SSGenEnv / DFGenEnv;

N = Ncol(data);

XX = char(N);

X= "X" + Strip(XX); Xs = "X1" : X;

O= shape(**0.001**, N, N);

OO=shape(**.**, N, N);

do i=**1** to N;

do j=**1** to N;

if Pvalue[i, j]=OO[i, j] then Pvalue[i, j]=OO[i, j];

else if O[i, j] >= Pvalue[i, j] then Pvalue[i, j]=o[i, j];

else if O[i, j] < Pvalue[i, j] then Pvalue[i, j]=Pvalue[i, j];

end;

end;

Var_Cov_G = (MSGen - MSGenEnv) / (Rep * Env);

Gen_var=vecdiag (Var_Cov_G)`;

print Gen_var [colname = Xs rowname="genotypic variance" label="these are the Genotypic Variance of the Traits"];

EGCov = (MSGenEnv - MSE) / Rep;

EnvCov = (MSE) + (EGCov / Rep);

EnvVar = (vecdiag (EnvCov))`;

PhenVar = (vecdiag (Var_Cov_G))` + EnvVar;

H = (vecdiag (Var_Cov_G))` / PhenVar * **100**;

SeH = (((Gen_var)##(**1**/**2**)) / PhenVar) * **100**;

print EnvVar [colname=Xs rowname="Environmental variance" label="These are the evironmental variance of the traits"];

print PhenVar [colname=Xs rowname="Phenotypic variance" label="These are the phenotypic variance of the traits"];

print H [colname=Xs rowname="Heritability" label= "These are the heritability of the traits"];

Print SEH [colname=Xs rowname="Standard error for herittability" label="These are the standard error for heritability of the traits"];

GCV =(sqrt( vecdiag (Var_Cov_G)))` / mean ***100**;

PCV = sqrt(PhenVar) / Mean * **100**;

print PCV [colname=Xs rowname="PCV" label="These are the phenotypic coefficient of variation for the traits"];

print GCV[colname=Xs rowname="GCV" label="These are the genotypic coefficient of variation for the traits"];

Resp = **2.06** # (sqrt(PhenVar)) # (H/**100**);

M_Next = Mean + Resp;

print Resp [colname=Xs rowname="response to selection" label="these are response of the traits for selection in current generation by screening 5% of extrem endividuals"];

print Mean[colname=Xs label="these are the mean of the traits for current generatin"];

Print M_Next[colname=Xs rowname="next generation mean" label="these are Mean of the next generation in response to selection in current generation

by screening 5% of extrem endividuals"];

**QUIT**;
